# Cell polarisation in a bulk-surface model can be driven by both classic and non-classic Turing instability

**DOI:** 10.1038/s41540-021-00173-x

**Published:** 2021-02-26

**Authors:** Johannes Borgqvist, Adam Malik, Carl Lundholm, Anders Logg, Philip Gerlee, Marija Cvijovic

**Affiliations:** Department of Mathematical Sciences, Chalmers University of Technology and the University of Gothenburg, Gothenburg, Sweden

**Keywords:** Numerical simulations, Dynamical systems

## Abstract

The GTPase Cdc42 is the master regulator of eukaryotic cell polarisation. During this process, the active form of Cdc42 is accumulated at a particular site on the cell membrane called the *pole*. It is believed that the accumulation of the active Cdc42 resulting in a pole is driven by a combination of activation–inactivation reactions and diffusion. It has been proposed using mathematical modelling that this is the result of diffusion-driven instability, originally proposed by Alan Turing. In this study, we developed, analysed and validated a 3D bulk-surface model of the dynamics of Cdc42. We show that the model can undergo both classic and non-classic Turing instability by deriving necessary conditions for which this occurs and conclude that the non-classic case can be viewed as a limit case of the classic case of diffusion-driven instability. Using three-dimensional Spatio-temporal simulation we predicted pole size and time to polarisation, suggesting that cell polarisation is mainly driven by the reaction strength parameter and that the size of the pole is determined by the relative diffusion.

## Introduction

*Cell division control protein 42 homologue*, *Cdc42* is an enzyme of the class GTPases that regulates various signalling pathways involved in cell division and cell morphology^1,2^. It is one of the most conserved GTPases where the Cdc42 in yeast is 80% identical to that in human cells^3^. In the late G_1_-phase during the cell cycle, a sequence of events causes the accumulation of Cdc42^4^, which is the master regulator of cell division, at a specific location on the cell membrane. This location is called the *pole* which is the site where the new cell grows out and the latter process is called budding in the case of the yeast *Saccharomyces cerevisiae*. Moreover, in the cytosol Cdc42 is bound to GDP which corresponds to its inactive state while it is bound to GTP in the membrane corresponding to its active form. The conversion between these two states is catalysed by the two classes of enzymes called GEFs and GAPs. It is believed that it is the combination of these reactions of activation and inactivation along with diffusion that results in the accumulation of active Cdc42.

Experimentally, the challenge with studying the activation of Cdc42 is that the concentration profile is not uniform in the cell. Thus, accounting for spatial inhomogeneities is crucial when data of the pathway is collected, however, measuring two different diffusion rates simultaneously is difficult. Firstly, measuring the slow diffusion rate of active Cdc42 on the cell membrane is not trivial. Secondly, in addition to accounting for the spatial distribution of Cdc42, the activation and inactivation reaction rates should be measured as well. Usually, such rates are estimated from data of spatial averages of the concentration profiles over time which is perhaps not feasible to do in the case of the mentioned polarisation system as inhomogeneous distributions of proteins are crucial for the function of the system. On account of these experimental limitations, computational models have been developed to aid in understanding the activity of Cdc42.

Numerous mathematical models of the dynamics of Cdc42 have been developed^5–18^. Many of these models can be reduced to a classic *activator–inhibitor* system focusing on the spatial and temporal dynamics of active and inactive Cdc42^5,8,10,12,15–17^. An important consideration in such models is whether the accumulation of active Cdc42 in a single location is the result of a Turing-type mechanism or not. Early models of polarisation have a single spatial dimension describing either the chemical concentration along a diameter of the cell or the cell perimeter while considering the cytosol as spatially uniform. Later, however, a model on the single-cell scale was developed where Turing patterns formed on the cell membrane in the presence of non-linear reactions involving another species diffusing in the cytosol^19^. It was demonstrated that with this new type of bulk-surface model, a distinct type of pattern formation mechanism was possible. In classic Turing-type systems, equal diffusion rates of the reacting species can never produce patterns, however, this is no longer a necessary restriction in the bulk-surface model. It has been argued that the necessary difference in transport can be achieved by choosing the various reaction rates to be unequal and this is referred to as non-classic Turing patterns^20,21^. Sufficient conditions for the emergence of both classic and non-classic Turing patterns in the context of bulk-surface models have been derived and demonstrated^20,21^. Additionally, a previous one-dimensional model of cell polarisation^14^ has been extended to the bulk-surface context^22^. This model is a two-species system of active and inactive GTPases, and does not distinguish between the inactive form in the cytosol and the inactive form in the membrane. An effort has been made to extend classical 1D models into 2D^8^ as well as including a number of more complicated phenomena such as a diffusion barrier on the membrane, and the presence of organelles in the cytosol.

Although bulk-surface models of polarisation is not a novel concept, most previous work has been focused on the occurrence of pattern formation and the qualitative behaviour of the models. However, little has been done in order to investigate the parameter space and the regions that give rise to classic or non-classic Turing patterns. In this work, we constructed a reaction–diffusion model of Cdc42 activation with the aim to propose an underlying mechanism of cell polarisation. We use mathematical analysis to investigate the two cases of classic and non-classic diffusion-driven instability. Moreover, we conducted an analysis of the parameter space and investigated how it influences polarisation. With these results in hand, we derived a necessary condition for diffusion-driven instability and showed using numerical simulations that the model can form patterns through both classic and non-classic Turing instability. Finally, we validated the proposed mechanisms using three-dimensional Spatio-temporal simulations of the developed model. This resulted in precise conditions allowing for the formation of a single pole. Also, this enabled us to determine how the involved parameters influence the time to polarisation, size of the pole as well as the local concentration of active Cdc42 at the pole.

## Results

### The reaction–diffusion model of Cdc42 activation

**Fig. 1:**
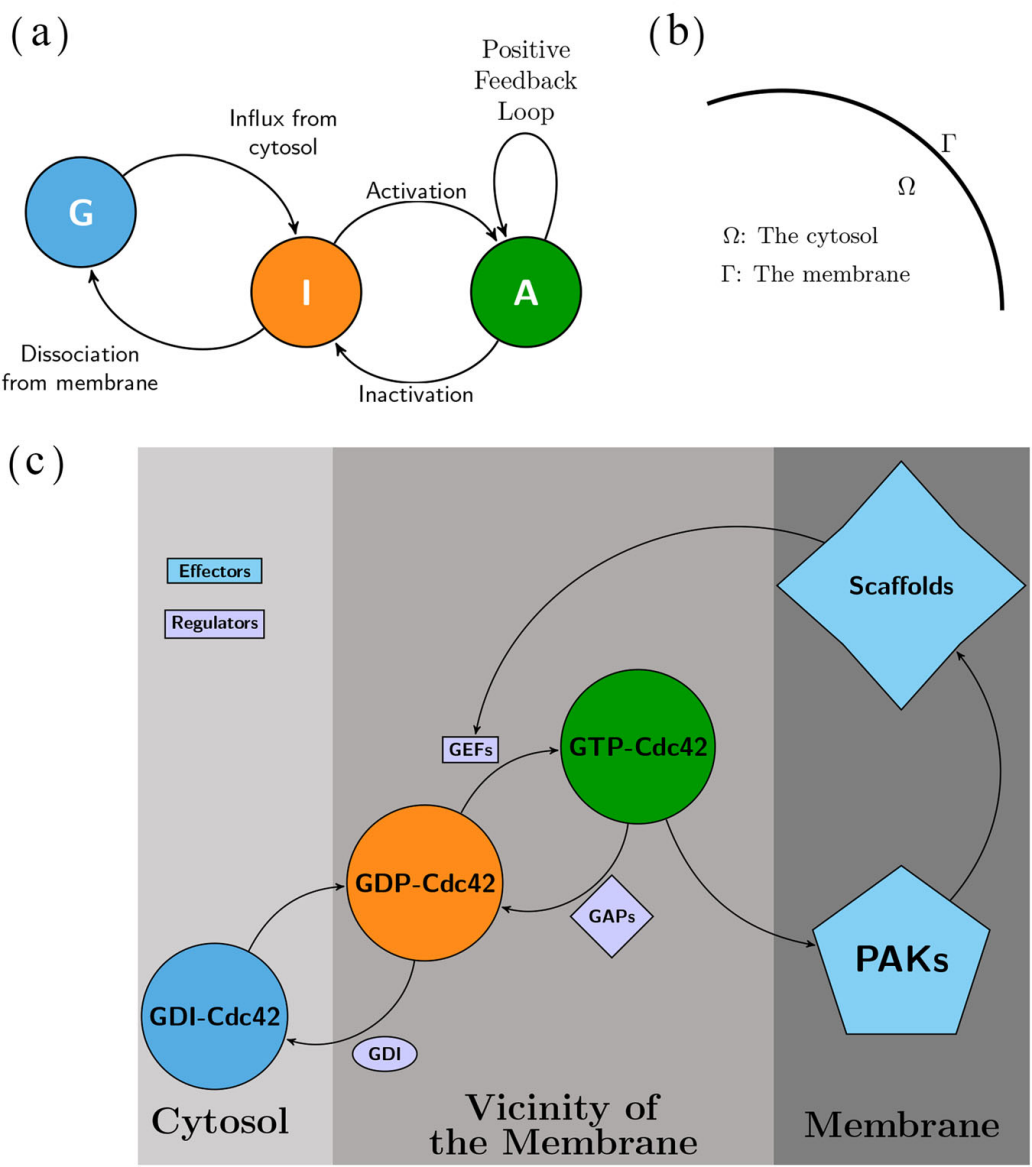
The model of Cdc42-activation. **(a)** *The mechanism described as an expanded activator–inhibitor system*. The GTPase Cdc42 is shuffled between its active state *A* (green) corresponding to the GTP-bound form and its inactive state *I* (orange) corresponding to the GDP-bound form. Also, the GDI-bound form *G* (blue) is restricted to the cytosol and is transported to the membrane which corresponds to the influx of inactive form *I*. The reaction mechanism is determined by three classes of reactions: (1) Flux of inactive Cdc42 over the membrane, (2) Activation and inactivation reactions restricted to the membrane determined by GEFs and GAPs respectively and (3) The positive feedback loop mediated by PAKs and Scaffolds also restricted to the membrane. **(b)** *Geometric domain*. By letting the membrane thickness shrink to zero, the geometric description is simplified to one domain Ω corresponding to the cytosol and one boundary Γ corresponding to the membrane. **(c)** *Biological details of Cdc42 activation*. Cdc42 has an inhibited GDI-bound form (blue), an inactive GDP-bound form (orange) and an active GTP-bound form (green). The shuffling between these forms is determined by the three regulators namely GDI, GEFs and GAPs. The active form of Cdc42, unlike the inactive, can bind to various effector molecules such as PAKs and Scaffolds which can further bind to GEFs which enhances the activation reaction through a positive feedback loop. This sub panel is re-drawn based on schematic representations in^9,25^.

To derive the reaction mechanism for the polarisation process mediated by Cdc42, we describe the cell as the interior of a three-dimensional ball Ω with radius *R* where its surface Γ corresponds to the cell membrane (Fig. 1b):

1 $$\begin{array}{ll}{{\Omega }}\,:=\left\{{\bf{x}}\in {{\mathbb{R}}}^{3}:| | {\bf{x}}| {| }_{2}\,<\, R\right\}\\ {{\Gamma }}\,:=\left\{{\bf{x}}\in {{\mathbb{R}}}^{3}:| | {\bf{x}}| {| }_{2}=R\right\}\end{array}$$

where ∣∣**x**∣∣_2_ is the Euclidian distance measure. As the cytosol Ω is comparatively large compared to the membrane it could be considered as a bulk which is subsequently linked to the two-dimensional membrane Γ. We refer to models which account for this geometric description (1) as *bulk-surface* models. In this way, it is possible to expand the classic Turing framework^23^ to account for reaction-diffusion models with two geometric domains which are both the most natural and more realistic than one-dimensional simplifications in the context of Cdc42.

Using this notation, we derive a *bulk surface Activator–Inhibitor* (*AI*) system of cell polarisation mediated by Cdc42 whose dynamics is governed by five reactions: *Influx of inactive Cdc42 from the cytosol*, *dissociation of inactive Cdc42 from the membrane*, *activation of inactive Cdc42*, *inactivation of active Cdc42* and *activation of Cdc42 through a positive feedback loop* (Fig. 1 and Supplementary Text 1.1):

2 $$\begin{array}{ll}{\mathrm{Influx}}\,{\mathrm{rate}}\ \ \ \ \ \ \ \ \ \ \ \ \ \ \ \ \ \ \ \ \ \ \ \,={k}_{1}\cdot G\cdot \left({k}_{\max }-(A+I)\right),\\ {\mathrm{Dissociation}}\,{\mathrm{rate}}\ \ \ \ \ \ \ \ \ \ \ \ \,={k}_{-1}\cdot I,\\ {\mathrm{Activation}}\,{\mathrm{rate}}\ \ \ \ \ \ \ \ \ \ \ \ \ \ \ \,={k}_{2}\cdot I,\\ {\mathrm{Inactivation}}\,{\mathrm{rate}}\ \ \ \ \ \ \ \ \ \ \ \ \,={k}_{-2}\cdot A,\\ {\mathrm{Feedback}}\,{\mathrm{activation}}\,{\mathrm{rate}}\,={k}_{3}\cdot {A}^{2}\cdot I,\end{array}$$

where it is assumed that the rate parameters are non-negative, i.e. ${k}_{1},{k}_{-1},{k}_{\max },{k}_{2},{k}_{-2},{k}_{3}\,> \,0$. To this end, we introduce three functions

$$\begin{array}{l}G:{{\Omega }}\times [0,T]\to {\mathbb{R}}\\ A:{{\Gamma }}\times [0,T]\to {\mathbb{R}}\\ I:{{\Gamma }}\times [0,T]\to {\mathbb{R}}\end{array}$$

describing the concentrations of the GTP-, GDP- and GDI-bound forms, respectively. Both *G* and *I* are referred to as the inactive form of Cdc42, whereas *A* corresponds to the active form. The functions *A* and *I* are restricted to the membrane Γ while the function *G* is restricted to the cytosol Ω. The concentration in the cytosol have units in mol/m^3^, and the concentrations on the membrane have units in mol/m^2^.

Combining all these terms allows us to formulate a reaction–diffusion model for cell polarisation mediated by Cdc42:

3 $$\begin{array}{l}\frac{\partial G}{\partial t}={D}_{G}\Delta G, \,{\bf{x}}\in \Omega ,\ t\in{{\mathbb{R}}}_{+}\\ -{D}_{G}\left[{(\nabla G)}^{T}{\bf{n}}\right]={k}_{1}G\left({k}_{\max}-(A+I)\right)-{k}_{-1}I, \,{\bf{x}} \in \Gamma ,\ t\in{{\mathbb{R}}}_{+}\\ =Q(A,I,G),\\ \left.\begin{array}{ll}\frac{\partial A}{\partial t}&={k}_{2}I-{k}_{-2}A+{k}_{3}{A}^{2}I+{D}_{A}{\Delta }_{\Gamma }A\\ &=F(A,I)+{D}_{A}{\Delta }_{\Gamma }A\\ &\\ \frac{\partial I}{\partial t}&=-F(A,I)+Q(A,I,G)+{D}_{I}{\Delta }_{\Gamma }I\\ &=G(A,I)+{D}_{I}{\Delta }_{\Gamma }I\\ \end{array}\right\}, \,{\bf{x}}\in \Gamma ,\ t\in {{\mathbb{R}}}_{+}.\end{array}$$

Here, the diffusion is determined by the Laplace operator ${{\Delta }}=\mathop{\sum }\nolimits_{i = 1}^{n}({\partial }^{2}/\partial {x}_{i}^{2})$ where Δ_Γ_ determines the diffusion restricted to the membrane.

The dynamics in the cytosol is entirely described by the diffusion of the GDI-bound form *G*. The flux of the inactive GDI-bound form of Cdc42 *G* from the cytosol to the membrane resulting in the influx of the membrane-bound inactive GDP-bound form of Cdc42 *I* is determined by the function *Q*. The function *Q*(*A*, *I*, *G*) is implemented as a non-homogeneous Neumann boundary condition for the GDI-bound state *G* and it is also part of the reaction term for the membrane-bound GDP-bound state *I*. Moreover, the total mass of the system is conserved, independent of the choice of the functions *F* and *Q*, which can be seen by considering the temporal change of the total mass of the system, and using the partial differential equations it follows that

$$\begin{array}{ll}&\frac{{\rm{d}}}{{\rm{d}}t}\left({\int}_{{{\Omega }}}G({\bf{x}},t)\ {\rm{d}}{\bf{x}}+{\int}_{{{\Gamma }}}A({\bf{s}},t)+I({\bf{s}},t)\ {\rm{d}}{\bf{s}}\right)\\ &={\int}_{{{\Omega }}}{D}_{G}{{\Delta }}G\ {\rm{d}}{\bf{x}}+{\int}_{{{\Gamma }}}{D}_{A}{{{\Delta }}}_{{{\Gamma }}}A+Q(A,I,G)+{D}_{I}{{{\Delta }}}_{{{\Gamma }}}I\ {\rm{d}}{\bf{s}}\\ &={\int}_{{{\Gamma }}}{D}_{G}{(\nabla G)}^{T}{\bf{n}}+{D}_{A}{{{\Delta }}}_{{{\Gamma }}}A+Q(A,I,G)+{D}_{I}{{{\Delta }}}_{{{\Gamma }}}I\ {\rm{d}}{\bf{s}}\\ &={\int}_{{{\Gamma }}}{D}_{A}{{{\Delta }}}_{{{\Gamma }}}A+{D}_{I}{{{\Delta }}}_{{{\Gamma }}}I\ {\rm{d}}{\bf{s}}\\ &=0\end{array}$$

which implies that the total amount of protein is constant.

Furthermore, the model is non-dimensionalised using non-dimensional parameters similar to the ones in the classical Schnackenberg model^24^ (Supplementary Text 1.2), resulting in the following model structure:

4 $$\begin{array}{l}\frac{\partial V}{\partial t} =D{{\Delta }}V, \,{\bf{x}}\in {{\Omega}},\ t\in {{\mathbb{R}}}_{+}\\ -D\left[{(\nabla V)}^{T}{\bf{n}}\right]=\gamma\left\{{c}_{1}V\left({c}_{\max}-(u+v)\right)-{c}_{-1}v\right\}, {\bf{x}}\in {{\Gamma }},\ t\in {{\mathbb{R}}}_{+}\\ =\gamma q(u,v,V), \\ \left.\begin{array}{ll}\frac{\partial u}{\partial \tau }&=\gamma \left({c}_{2}v-u+{u}^{2}v\right)+{{{\Delta }}}_{{{\Gamma }}}u\\ &=\gamma f(u,v)+{{{\Delta }}}_{{{\Gamma }}}u\\ \frac{\partial v}{\partial \tau }&=\gamma \left(-f(u,v)+q(u,v,V)\right)+d{{{\Delta }}}_{{{\Gamma }}}v\\ \end{array}\right\},\ {\bf{x}}\in {{\Gamma }},\ t\in {{\mathbb{R}}}_{+}. \end{array}$$

Note here that in the dimensionless model, the domain Ω corresponds to the *unit ball* and the boundary Γ corresponds to the *unit sphere*.

As a result of the non-dimensionalisation procedure, the number of parameters has been reduced from ten to eight, where the resulting dimensionless parameters are also more meaningful compared to the original ones. For example, the parameter *γ* determines the relative strength of the *reaction* part of the model compared to the *diffusion* part which implies that this parameter determines which of these forces that dominate the dynamics of the system. Moreover, all of the dynamics corresponding to the activation, inactivation and the positive feedback loop is captured in the dimensionless parameter *c*_2_ which, in the case with dimensions, are described by the two parameters *k*_2_ and *k*_−2_. The dimensionless states, variables and parameters are summarised in Table 1.

**Table 1:** Dimensionless components of the model. The columns from left to right: the components (i.e. the states, variables and parameters), their definitions and a description of their meaning.

Component	Definition	Description
*The states*		
*u*	$u=A\cdot \sqrt{\frac{{k}_{3}}{{k}_{-2}}}$	The dimensionless active form of Cdc42 (*membrane-bound*)
*v*	$v=I\cdot \sqrt{\frac{{k}_{3}}{{k}_{-2}}}$	The dimensionless inactive form of Cdc42 (*membrane-bound*)
*V*	$V=\frac{G}{R}\cdot \sqrt{\frac{{k}_{3}}{{k}_{-2}}}$	The dimensionless GDI-bound Cdc42 (*cytosolic*)
*The variables*		
*τ*	$\tau =\frac{{D}_{A}t}{{R}^{2}}$	The dimensionless time (S14)
**x** ∈ Ω_*R*_	${\bf{x}}\leftarrow \frac{1}{R}\ {\bf{x}}$	The dimensionless spatial variable (S14)
*The parameters*		
*γ*	$\gamma =\frac{{R}^{2}{k}_{-2}}{{D}_{A}}$	The parameter has three interpretations [(ref. ^24^), page 78]:(**1**.) *γ* is proportional to the area of the domain Ω in (1). (**2**.) *γ* is the relative strength of the two reaction terms determined by *f*(*u*, *v*) and *q*(*u*, *v*) in (4). (**3**.) An increase in *γ* corresponds to an equivalent decrease in the diffusion coefficient *d*.
*c*_1_	${c}_{1}=\frac{{k}_{1}}{{k}_{-2}}\sqrt{\frac{{k}_{-2}}{{k}_{3}}}$	The relative influx of inactive Cdc42 from the cytosol
${c}_{\max }$	${c}_{\max }={k}_{\max }\sqrt{\frac{{k}_{3}}{{k}_{-2}}}$	The maximum amount of membrane-bound Cdc42, i.e. $(u+v)\le {c}_{\max }$
*c*_−1_	${c}_{-1}=\frac{{k}_{-1}}{{k}_{-2}}$	The relative dissociation of inactive Cdc42 from the membrane
*c*_2_	${c}_{2}=\frac{{k}_{2}}{{k}_{-2}}$	The relative activation rate of Cdc42
*d*	$d=\frac{{D}_{I}}{{D}_{A}}$	The relative diffusion rate of inactive Cdc42 (*diffusion in the membrane* Γ)
*D*	$D=\frac{{D}_{G}}{{D}_{A}}$	The relative diffusion rate of GDI-bound Cdc42 (*diffusion in the cytosol* Ω)
*V*_0_	${V}_{0}={G}_{0}\cdot \sqrt{\frac{{k}_{3}}{{k}_{-2}}}$	The initial amount of dimensionless GDI-bound Cdc42

As the cytosolic GDI-bound state of Cdc42 diffuses much faster than the membrane bound states^25^, it is possible to reduce the number of equations in (4). More precisely, the assumption that *D* → *∞* implies that the concentration of the cytosolic GDI-bound state is homogeneous and in this case the mass conservation property is described by the *non-local functional* *V*[*u* + *v*] below

5 $$V[u+v]={V}_{0}-\frac{1}{| {{\Omega }}| }{\int_{\Gamma }}(u+v)\ {\rm{d}}s$$

and the RD-system in (4) gets reduced to the following two-state system

6 $$\left.\begin{array}{l}\frac{\partial u}{\partial \tau }={{\Delta }}u+\gamma f(u,v)\\ \frac{\partial v}{\partial \tau }=d{{\Delta }}v+\gamma (-f(u,v)+q(u,v,V[u+v]))\\ \end{array}\right\},\ {\bf{x}}\in {{\Gamma }},\ t\in {{\mathbb{R}}}_{+}.$$

The constant *V*_0_ is the total average concentration of all three forms of Cdc42, and *V*_0_∣Ω∣ is the total amount of Cdc42 in the cell. The full system in (4) is solved numerically while the reduced system in (6) is analysed to determine if Turing patterns can be formed.

The model presented here builds on the framework proposed by Ratz and Roger^21,26^, where the function *f* describing the dynamics of the activation, inactivation and feedback loop of Cdc42 assumes *Michaelis–Menten* kinetics and is given by:

7 $$f(u,v)=\left({a}_{1}+({a}_{3}-{a}_{1})\frac{u}{{a}_{2}+u}\right)v-{a}_{4}\frac{u}{{a}_{5}+u}.$$

In (7), the dimensionless parameters *a*_1_, *a*_2_, *a*_3_, *a*_4_ and *a*_5_ correspond to kinetic parameters, where the kinetics is modelled by the law of mass action kinetics and a Michaelis–Menten term for modelling the enzymatically catalysed reactions, and it is worth mentioning that this reaction term is similar to the one described in^10^. In the context of the Cdc42 model, the *Michaelis–Menten* assumption implies that the substrates would be the various states of Cdc42 while the enzymes would be the GEF’s and GAP’s. However, as Cdc42 itself is an enzyme it is more reasonable to assume that its intracellular concentration is in the same order of magnitude as that of the GAP’s and the GEF’s. Therefore, in contrast to^21,26^, we describe the dynamics of Cdc42 governed by the activation, inactivation and the feedback loop with the simpler structure

$$f(u,v)={c}_{2}v-u+{u}^{2}v.$$

Here, the parameter *c*_2_ describing the relative activation rate of Cdc42 is well-motivated by the literature (Fig. 1). In contrast to the previous framework involving a larger number of parameters also resulting from a non-dimensionalisation although a different one^21,26^, the simplicity of our reaction term ensures that our approach can qualitatively model cell polarisation as each parameter has a concrete meaning. For example, a large value of the parameter *c*_2_ relative to the value of *c*_−1_ implies a high activity of the activation–inactivation module monitored by GEF’s, GAP’s and the positive feedback loop relative to the dissociation from the membrane implying the attachment of GDIs to the inactive form of Cdc42.

### The bulk-surface model can form patterns through both classic and non-classic Turing patterns

Given the derived model, we can answer three fundamental questions. Does a unique solution to the RD system in (6) determined by the initial conditions exist? If so, are the solutions physically realistic, meaning that they give rise to non-negative and bounded concentrations of *u* and *v*? If this is the case, can the model undergo diffusion-driven instability and thereby form patterns? To answer these questions, we define the *homogeneous* system of the reduced model in (6) as follows

8 $$\begin{array}{ll}\frac{{\rm{d}}\overline{u}}{{\rm{d}}\tau }\,=\gamma f(\overline{u},\overline{v})\\ \frac{{\rm{d}}\overline{v}}{{\rm{d}}\tau }\,=\gamma (f(\overline{u},\overline{v})-{q}_{0}(\overline{u},\overline{v}))\\ \end{array}$$

where the states $\overline{u},\overline{v}$ are the spatial averages of *u* and *v* respectively. Here, the function ${q}_{0}(\overline{u},\overline{v})={c}_{1}a({c}_{\max }-(\overline{u}+\overline{v}))(m-(\overline{u}+\overline{v}))-{c}_{-1}\overline{v}$ where *m* = *V*_0_/*a* is obtained as a consequence of the spatial averaging as the functional *V* in (5) is replaced by $\overline{V}={V}_{0}-(| {{\Gamma }}| /| {{\Omega }}| )(\overline{u}+\overline{v})={V}_{0}-a(\overline{u}+\overline{v})$ where *a* = 3 is the ratio between the area of the unit sphere divided by the volume of the unit ball. Moreover, as we are interested in non-negative states, we require that $\left(\overline{u}+\overline{v}\right)\le {V}_{0}/a=m$ in (8) which implies $0\le \overline{V}={V}_{0}-a(\overline{u}+\overline{v})$ where the expression for the cytosolic component $\overline{V}$ stems from the mass conservation functional. In addition, the total amount of membrane-bound proteins is also constrained by ${c}_{\max }$, i.e. $\left(\overline{u}+\overline{v}\right)\le {c}_{\max }$. Therefore, physically reasonable states corresponding to $\overline{u},\overline{v},\overline{V}\ge 0$ lie in the region ${\mathcal{A}}$ in the (*u*, *v*)-state space defined as follows

9 $${\mathcal{A}}:=\left\{\left(\begin{array}{l}u\\ v\end{array}\right)\ge \left(\begin{array}{l}0\\ 0\end{array}\right):u+v\le \min \left\{{c}_{\max },m\right\}\right\}\ \ {\rm{where}}\ m=\frac{{V}_{0}}{a}.$$

#### Existence of a unique solution

The existence of solutions to RD models is not guaranteed for all continuous reaction functions *f* and *q*. To this end, we prove the existence of a unique solution (Thm 1) to the reduced model in (6) if we choose the initial conditions from the previously defined region ${\mathcal{A}}$ in (9) (Supplementary Text 1.3).

##### Theorem 1

(**Existence of a unique solution on the unit sphere in global time**). Consider the RD model in (6) with initial conditions *u*(**x**, 0) = *u*_0_(**x**) and *v*(**x**, 0) = *v*_0_(**x**) chosen such that $({u}_{0}({\bf{x}}),{v}_{0}({\bf{x}}))\in {\mathcal{A}}\ \forall {\bf{x}}\in {{\Gamma }}$ where the region ${\mathcal{A}}$ is defined in (9) and where the spatial derivatives (in a weak sense) of the initial conditions are bounded in the ${{\mathcal{L}}}_{2}({{\Gamma }})$ norm, i.e. $| | {\nabla }_{{{\Gamma }}}{u}_{0}| {| }_{{{\mathcal{L}}}_{2}({{\Gamma }})}\,<\,\infty$ and $| | {\nabla }_{{{\Gamma }}}{v}_{0}| {| }_{{{\mathcal{L}}}_{2}({{\Gamma }})}\,<\,\infty$. Then, there exists a unique solution to (6) global in time.

#### Physical validity of the model

We have already established one physical property, namely mass conservation, which governs the system. However, this property follows from the structure of the model implying that any choice of the reaction terms *f* and *q* results in a model with this property. Also, the fact that the mass of the states *u*, *v* and *V* is conserved does not prevent non-physical behaviour of the solutions such as negative concentrations of the involved proteins, e.g. *u*(**x**, *τ*) ≤ 0 for some coordinate **x** ∈ Γ at some time *τ*. To this end, we prove (Thm 2) that the region ${\mathcal{A}}$ in (9) is a *trapping region* meaning that if the initial conditions are chosen within this region then we will always have non-negative solutions at all times to the homogeneous system (8), but not to the full PDE problem (6) (Supplementary Text 1.4). Interestingly enough, from this follows another theoretical result (Corollary 1) which states that the domain Γ can deviate from the unit sphere and we will still have a unique solution to the RD model in (6) global time (Supplementary Text 1.4.1). The latter result implies that with our model, i.e. choice of *f* in combination with *q*, the shape of the membrane can deviate from the sphere and the model will still have a solution that is uniquely determined by its initial conditions.

##### Theorem 2

(**Existence of a trapping region**). The region ${\mathcal{A}}\subset {{\mathbb{R}}}^{2}$ in (9) is a trapping region meaning that the trajectories of the solutions to the homogeneous system in (8) can never leave the region for initial conditions $(u(0),v(0))=({u}_{0},{v}_{0})\in {\mathcal{A}}$.

##### Corollary 1

(**Existence of a unique solution on any open bounded regular subset of** ${{\mathbb{R}}}^{n}$ **in global time**). Consider the RD model in (6) where the domain ${{\Gamma }}\subset {{\mathbb{R}}}^{n}$ for any $n\in {{\mathbb{N}}}_{+}$ is changed to an open, bounded and regular domain with Neumann (i.e. zero-flux) boundary conditions. If the initial conditions *u*(**x**, 0) = *u*_0_(**x**) and *v*(**x**, 0) = *v*_0_(**x**) are chosen such that $({u}_{0}({\bf{x}}),{v}_{0}({\bf{x}}))\in {\mathcal{A}}\ \forall {\bf{x}}\in {{\Gamma }}$ where the region ${\mathcal{A}}$ is defined in (9), then this problem has a unique solution global in time.

#### Diffusion-driven instability

The underlying idea behind pattern formation caused by diffusion-driven instability pattern formation was originally formulated by Turing^23^, and it entails a switch in stability in the sense of linear stability analysis. The phenomena depend on the reaction terms, e.g. *f* and *q*, having the capacity to allow for the existence of a stable steady state (u^*^, v^*^) to the homogeneous-ODE system obtained by neglecting diffusion. When diffusion is introduced, *classic* diffusion-driven instability occurs when a *stable node* in the homogeneous system is switched to an *unstable node* in the inhomogeneous system. In the *non-classic* diffusion-driven instability^21,26^ this takes place when a steady-state (u^*^, v^*^) transitions from a *stable node* in the homogeneous system to a *saddle point* in the inhomogeneous system. The exact formulation of the mathematical conditions for these two cases is presented in the Supplementary Text 1.5. It is important to emphasise that both cases rely on the existence of a steady state with the capacity of switching stability, and it is thus crucial to find rate parameters ensuring this fundamental requirement. To this end, we have mathematically proven that the model has steady states, and we have derived a necessary condition ensuring the stability of a specific steady state (Thm 3) (Supplementary Text 1.6).

##### Theorem 3

(**Existence and characterisation of steady states**). The system in (8) with non-negative rate parameters ${c}_{1},{c}_{-1},{c}_{2}\ {\rm{and}}\ {c}_{\max }$ has either 0, 2, 4 or 6 positive steady-states within the first rant of the (*u*, *v*)-state space. In fact, the non-negativity of the parameters is a sufficient condition for ensuring the existence of at least one steady-state $({u}^{* },{v}^{* })\in {\mathcal{A}}$ in the trapping region in (9). Lastly, a steady-state $({u}^{* },{v}^{* })\in {\mathcal{A}}$ allowing for diffusion-driven instability satisfies the following necessary condition:

10 $$\sqrt{{c}_{2}}\,<\,\min \left\{{c}_{\max },m\right\}\ \ {\rm{and}}\ \ {u}^{* }\in \left(\sqrt{{c}_{2}},\min \left\{{c}_{\max },m\right\}\right).$$

The necessary condition in (10) implies that the activation-inactivation parameter *c*_2_ is constrained by the maximum concentration of membrane-bound species, ${c}_{\max }$, and the average total concentration of cytosolic Cdc42, *V*_0_. Furthermore, provided that we restrict ourselves to non-negative rate parameters, there is always one steady-state (possibly more) in the trapping region in (9). Combining this restriction with the bound in (10) in Thm 3, this yields a characterisation of a steady-state (u^*^, v^*^) which could potentially give rise to patterns and that is that it always lies in the interval ${u}^{* }\in (\sqrt{{c}_{2}},\min \left\{{c}_{\max },{V}_{0}/a\right\})$. These bounds indicate that using the rate parameter *c*_2_, corresponding to the activation-inactivation reactions, it is possible to formulate a lower bound on the steady-state. Combining the maximum concentration of membrane-bound species ${c}_{\max }$ with the total average concentration *V*_0_ of the cytosolic state the upper bound can be formulated. Note that these conditions merely ensure the existence of steady-states, and to ensure the emergence of patterns the exact steady-state satisfying the mathematical conditions in the stability analysis must be found.

### Numerical mappings of the parameter space indicate that the non-classic case of diffusion-driven instability is a limiting class of the classic case

**Fig. 2:**
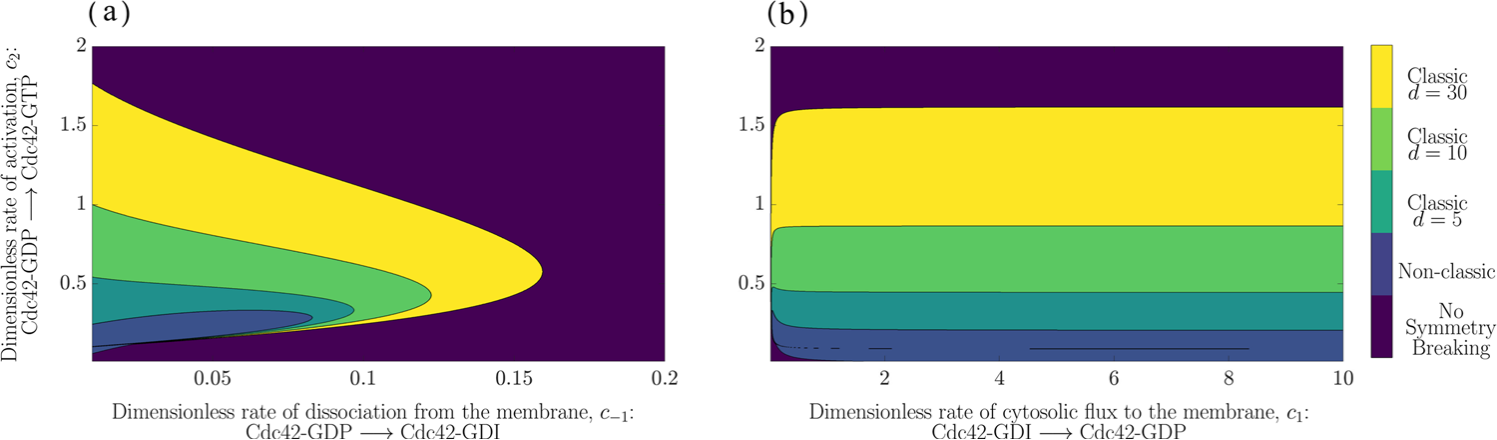
The parameter space for classic and non-classic diffusion-driven instability. The parameter space is divided into five regions indicated by the colour bar: *Classic Turing instability* with *d* = 30 (yellow), *Classic Turing instability* with *d* = 10 (light green), *Classic Turing instability* with *d* = 5 (green blue), *Non-classic Turing instability* (light blue) and *No Symmetry Breaking* (dark blue). **(a)** the (*c*_−1_, *c*_2_)—plane with a fixed value of *c*_1_ = 0.05. **(b)** the (*c*_1_, *c*_2_)—plane with a fixed value of *c*_−1_ = 0.05. The overall parameters in both cases are *V*_0_ = 6.0 and ${c}_{\max }=3.0$ (note that the non-classic case is independent of *d*).

Next, we investigate the steady-states by numerical exploration of the parameter space to determine whether they satisfy the classic or non-classic Turing conditions derived by Roger and Ratz^21,26^. To this end, we investigated a large set of kinetic parameters by mapping out the (*c*_−1_, *c*_2_) and (*c*_1_, *c*_2_) cross sections of the parameter space. For each point in these cross sections, two kinetic parameters are varied while the remaining parameters are kept fixed. We observed that for a large relative diffusion, *d*, the set of parameters enabling pattern formation is larger in the classic than the non-classic case (Fig. 2). It is worth emphasising that the region where the Turing conditions are met for *d* = 5, is a subset of the region for any larger *d* and in the figures, we have chosen to plot the regions in layers, with the lowest *d* shown on top. Also, the non-classic region is special in the sense that the classical Turing conditions can never be met when *d* = 1. This implies that the set of parameters that allow for symmetry breaking increases with the relative diffusion, *d*, in both cases of varying (*c*_−1_, *c*_2_) and (*c*_1_, *c*_2_) (Fig. 2). Conversely, when the relative diffusion decreases the region of the parameter space allowing for diffusion-driven instability decreases as well, and in fact, the non-classic case can be viewed as the classic case in the limit *d* → 1. Note here, that the non-classic case is independent of the relative diffusion *d* and in the case where *d* = 1 the system can only form patterns through non-classic diffusion-driven instability. However, as soon as *d* > 1 symmetry breaking can occur through both mechanisms and when the relative diffusion is large the classic region of the parameter space in larger than the non-classic counterpart.

Further, we observe that the relative activation rate *c*_2_ must be larger than the relative dissociation rate *c*_−1_ in order to allow classic diffusion-driven instability (Fig. 2a). For all tested values of the relative diffusion, the relative activation rate *c*_2_ is approximately five to ten times larger than *c*_−1_. Additionally, within a range of relative activation rates *c*_2_ the phenomena of diffusion-driven instability is *independent* of the relative influx rate *c*_1_ (Fig. 2b). This conclusion holds true for larger values of the relative cytosolic flux than *c*_1_ = 10 although the parameter space is only illustrated up to this value. Note that a general result from both parameter planes is that classic Turing instability occurs for higher relative activation rates *c*_2_ compared to the non-classic case.

Provided these theoretical results, we next modelled cell polarisation using numerical simulations. We are interested in a specific pattern namely the formation of a single pole corresponding to a single circular spot of active Cdc42 on the cell membrane.

### Cell polarisation can be modelled through both classic and non-classic Turing patterns

**Fig. 3:**
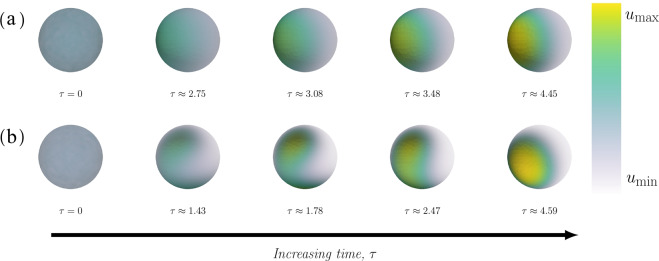
The time evolution of a pattern. The time evolution of the concentration profiles for two sets of parameters corresponding to classic and non-classic, respectively. **(a)** *Classic*: The parameters are (*c*_−1_, *c*_2_) = (0.02, 0.45) where the time points, from left to right, are *τ* = 0, *τ* ≈ 2.75, *τ* ≈ 3.08, *τ* ≈ 3.48 and *τ* ≈ 4.45. The maximal and minimal concentration defining the bounds on the colour bar is given by $({u}_{\min },{u}_{\max })=(0.38,3.77)$ in the classic case. **(b)** *Non-classic*: The parameters are (*c*_−1_, *c*_2_) = (0.01, 0.20) where the time points, from left to right, are *τ* = 0, *τ* ≈ 1.43, *τ* ≈ 1.78, *τ* ≈ 2.47 and *τ* ≈ 4.59. The maximal and minimal concentration defining the bounds on the colour bar is given by $({u}_{\min },{u}_{\max })=(0.15,4.26)$ in the non-classic case. In both cases, the overall parameters are: *c*_1_ = 0.05, *V*_0_ = 6.0, ${c}_{\max }=3.0$, *a* = 3, *d* = 10 and *γ* = 25.

Cell polarisation can be modelled by both cases of diffusion driven instability (Fig. 3). Although the time evolution of the concentration profiles is slightly different, the final patterns are qualitatively very similar for the two cases. The classic case (Fig. 3a) forms a circular pole directly while the non-classic case (Fig. 3b) initially forms an elongated pattern which gradually transitions into a pole. Given that the model can simulate cell polarisation, we further investigate the impact of the kinetic rate constants *c*_1_, *c*_−1_ and *c*_2_, the relative diffusion *d* and the reaction strength *γ* on cell polarisation. To this end, we define three quantitative measures of polarisation: the size of the pole, the time to polarisation as well as the maximum and minimum concentration of active Cdc42 in order to quantify the effect of altering the various parameters. In the interest of comparing the previously mentioned measures between different cases of diffusion-driven instability as well as for different sets of parameters, we have implemented a "pole recognition” algorithm (Supplementary Text 2.3) which terminates the simulation when a pole has been formed.

### The effect of the relative influx, the disassociation and activation rates of Cdc42 on cell polarisation

The final patterns for different kinetic parameters are qualitatively similar but quantitatively different (Supplementary Fig. 4). Both the classic (Supplementary Fig. 4a) and the non-classic (Supplementary Fig. 4b) case form a single pole for different parameters within the (*c*_−1_, *c*_2_)-space (Fig. 2a). However, from a quantitative perspective the time it takes to form a pole, *τ*_final_, differs for different sets of kinetic parameters. For instance, in the classic case, it varies from *τ*_final_ ≈ 6.5 to *τ*_final_ ≈ 19.1 (Supplementary Fig. 4a), while in the non-classic case it varies from *τ*_final_ ≈ 2.88 to *τ*_final_ ≈ 9.0 (Supplementary Fig. 4b). Similarly, the maximum concentration of active Cdc42 ${u}_{\max }$ is different for different kinetic parameters. In the classic case, it varies from ${u}_{\max }=1.54$ to ${u}_{\max }=3.65$ (Supplementary Fig. 4a) while in the non-classic case it varies from ${u}_{\max }=3.86$ to ${u}_{\max }=4.02$ (Supplementary Fig. 4b). Similar conclusions can be drawn in the case of different parameters in the (*c*_1_, *c*_2_)-plane (Supplementary Fig. 5).

### The effect of increasing the relative diffusion on cell polarisation

**Fig. 4:**
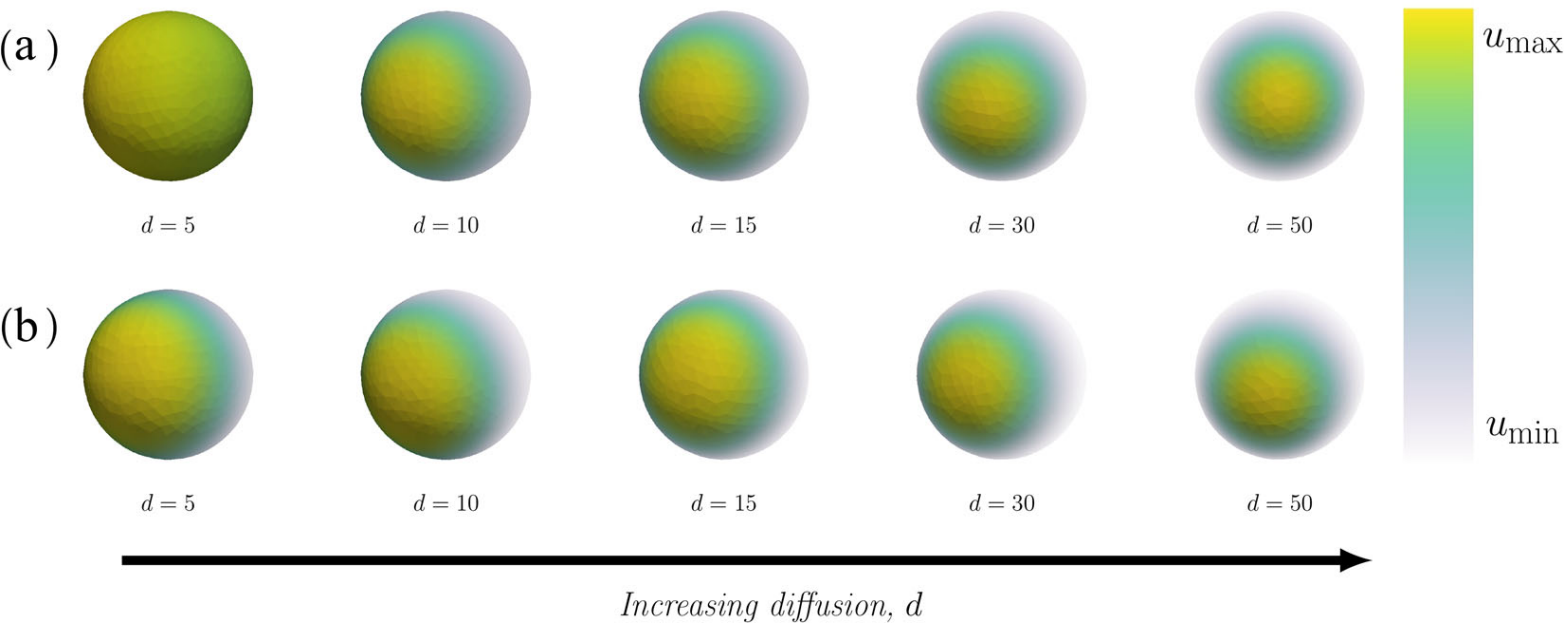
Final patterns for increasing relative diffusion with a relative scale. The final patterns for increasing relative diffusion *d* are displayed in two cases, namely classic and non-classic diffusion-driven instability. In both cases, the final time when the pattern is formed *τ*_final_ and the maximum and minimum concentrations of active Cdc42 ${u}_{\max }$ and ${u}_{\min }$ are calculated as functions of the kinetic rate parameters. **(a)** *Classic*: The overall parameters are (*c*_1_, *c*_−1_, *c*_2_) = (0.05, 0.04, 0.45) with specific parameters (from left to right): no pattern is formed for $(d,{\tau }_{{\rm{final}}},{u}_{\max },{u}_{\min })=(5,15,20.89,1.34,1.11)$, $(d,{\tau }_{{\rm{final}}},{u}_{\max },{u}_{\min })=(10,4.44,3.65,0.40)$, $(d,{\tau }_{{\rm{final}}},{u}_{\max },{u}_{\min })=(15,3.65,4.85,0.30)$, $(d,{\tau }_{{\rm{final}}},{u}_{\max },{u}_{\min })=(30,2.65,7.42,0.20)$ and $(d,{\tau }_{{\rm{final}}},{u}_{\max },{u}_{\min })=(50,2.17,9.83,0.15)$. **(b)** *Non-classic*: The overall parameters are (*c*_1_, *c*_−1_, *c*_2_) = (0.05, 0.03, 0.15) with specific parameters (from left to right): $(d,{\tau }_{{\rm{final}}},{u}_{\max },{u}_{\min })=(5,4.0,2.77,0.17)$, $(d,{\tau }_{{\rm{final}}},{u}_{\max },{u}_{\min })=(10,4.38,4.18,0.11)$, $(d,{\tau }_{{\rm{final}}},{u}_{\max },{u}_{\min })=(15,2.87,5.29,0.09)$, $(d,{\tau }_{{\rm{final}}},{u}_{\max },{u}_{\min })=(30,1.95,7.77,0.06)$ and $(d,{\tau }_{{\rm{final}}},{u}_{\max },{u}_{\min })=(50,1.96,10.166,0.04)$. In both cases, the overall parameters are: *V*_0_ = 6.0, ${c}_{\max }=3.0$, *a* = 3 and *γ* = 25.

**Fig. 5:**
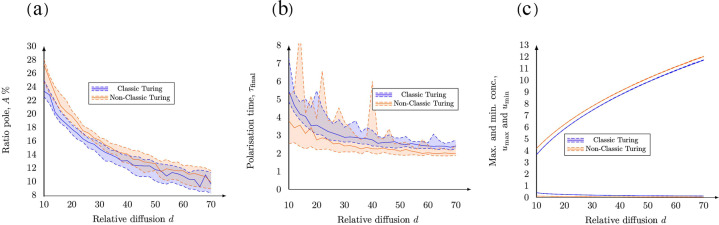
Quantitative measures as functions of an increasing relative diffusion *d*. The figure illustrates how the relative diffusion *d* influences (**a**) the size of the pole, (**b**) the time to polarisation and (**c**) the maximal and minimal values of *u* on the cell membrane. Due to the randomness in the initial conditions, the simulations have been run multiple times. Each data point on the curves corresponds to 20 realisations where the 95% (upper dashed line), 50% (full line) and 5% (lower dashed line) quantiles are plotted for each case, i.e. Classic and Non-classic Turing instability.

A single pole is formed in both the classic and non-classic case (Fig. 4) for all investigated cases of increasing relative diffusion *d*. An increase of the relative diffusion causes a decrease of the size of the pole, a decrease of the time to polarisation and an increase of the maximum (local) concentration of active Cdc42 in the pole (Fig. 5 and Supplementary Fig. 6). We did not observe any significant difference, neither qualitatively (Fig. 4) nor quantitatively (Fig. 5), between the two cases of diffusion-driven instability.

### The effect of increasing the relative reaction strength on cell polarisation

**Fig. 6:**
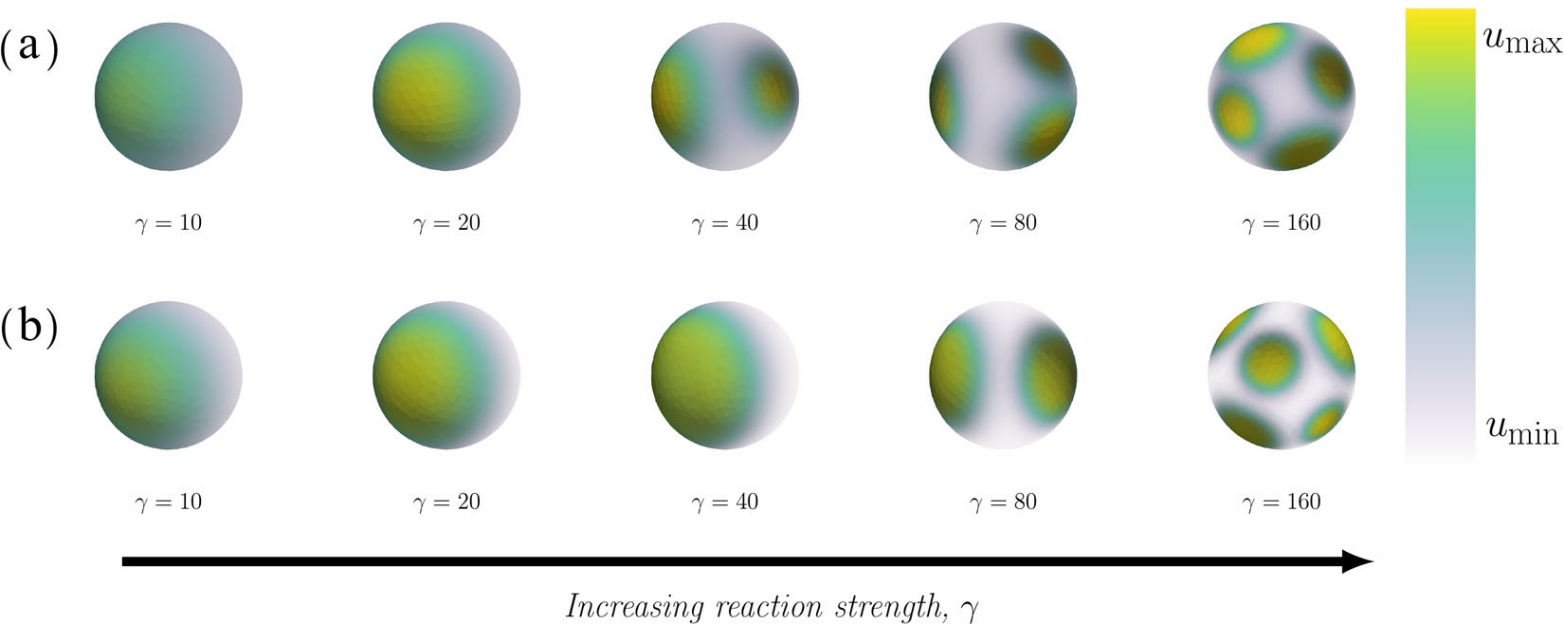
Final patterns for increasing relative reaction strength. The final patterns for increasing relative reaction strength *γ* are displayed in two cases, namely classic and non-classic diffusion-driven instability. In both cases, the maximum and minimum concentrations of active Cdc42 ${u}_{\max }$ and ${u}_{\min }$ are calculated as functions of the kinetic rate parameters. **(a)** *Classic*: The overall parameters are (*c*_1_, *c*_−1_, *c*_2_) = (0.05, 0.04, 0.45) with specific parameters (from left to right): $(\gamma ,{\tau }_{{\rm{final}}},{u}_{\max },{u}_{\min })=(10,10.81,3.02,0.49)$, $(\gamma ,{\tau }_{{\rm{final}}},{u}_{\max },{u}_{\min })=(20,5.06,3.62,0.40)$, $(\gamma ,{\tau }_{{\rm{final}}},{u}_{\max },{u}_{\min })=(40,5.86,3.75,0.44)$, $(\gamma ,{\tau }_{{\rm{final}}},{u}_{\max },{u}_{\min })=(80,4.63,3.80,0.40)$ and $(\gamma ,{\tau }_{{\rm{final}}},{u}_{\max },{u}_{\min })=(160,8.92,3.74,0.40)$. **(b)** *Non-classic*: The overall parameters are (*c*_1_, *c*_−1_, *c*_2_) = (0.05, 0.03, 0.15) with specific parameters (from left to right): $(\gamma ,{\tau }_{{\rm{final}}},{u}_{\max },{u}_{\min })=(10,4.66,3.96,0.15)$, $(\gamma ,{\tau }_{{\rm{final}}},{u}_{\max },{u}_{\min })=(20,2.77,4.21,0.11)$, $(\gamma ,{\tau }_{{\rm{final}}},{u}_{\max },{u}_{\min })=(40,4.41,4.00,0.11)$, $(\gamma ,{\tau }_{{\rm{final}}},{u}_{\max },{u}_{\min })=(80,11.28,4.15,0.12)$ and $(\gamma ,{\tau }_{{\rm{final}}},{u}_{\max },{u}_{\min })=(160,44,4.48,0.11)$. In both cases, the overall parameters are: *V*_0_ = 6.0, ${c}_{\max }=3.0$, *a* = 3 and *d* = 10.

**Fig. 7:**
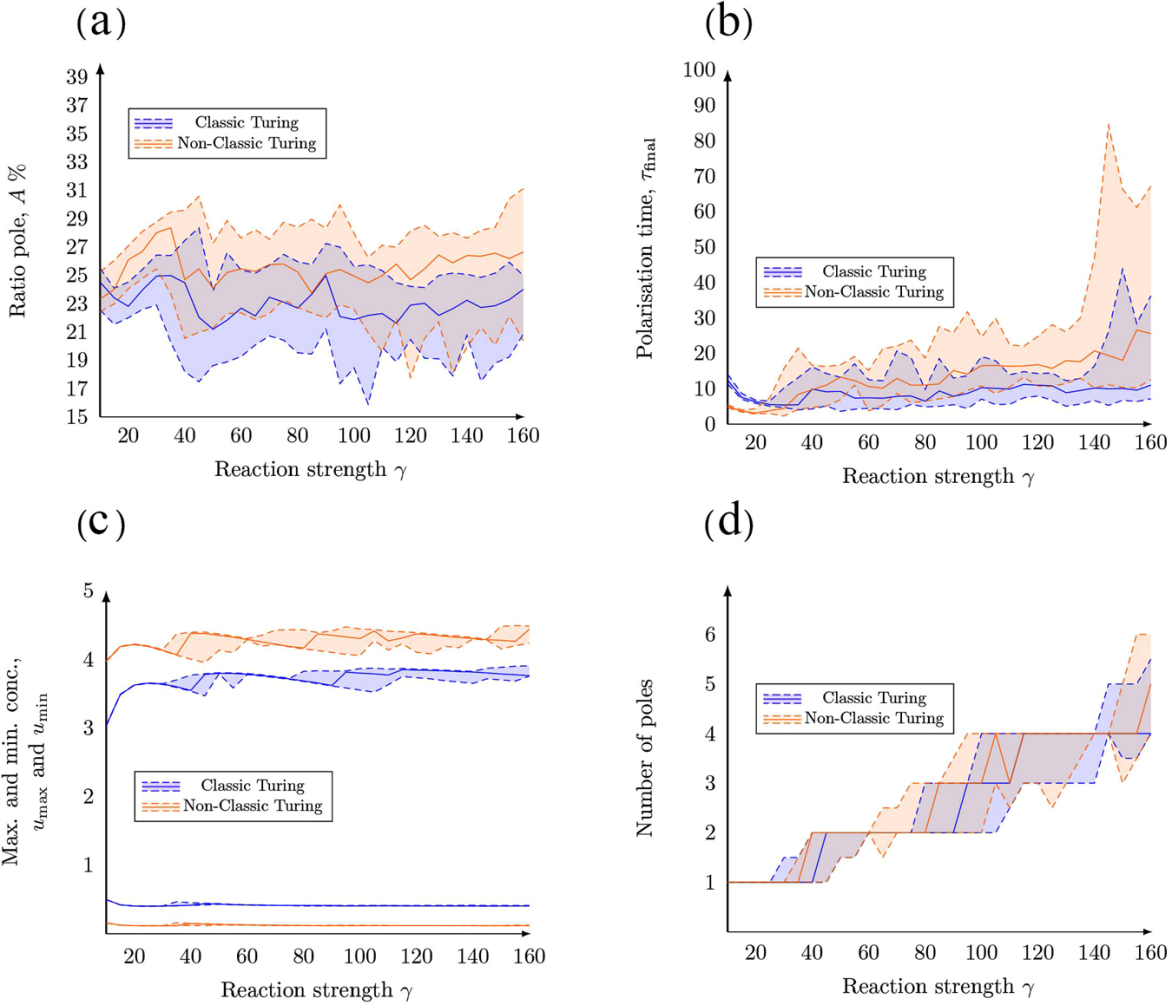
Quantitative measures as functions of increasing *γ*. The figure illustrates how the relative reaction strength *γ* influences **(a)** the size of the pole, **(b)** the time to polarisation, **(c)** the maximal and minimal values of *u* on the cell membrane, as well as **(d)** the number of poles. Due to the randomness in the initial conditions, the simulations have been run multiple times. Each data point on the curves corresponds to 20 realisations where the 95% (upper dashed line), 50% (full line) and 5% (lower dashed line) quantiles are plotted for each case, i.e. Classic and Non-classic Turing instability.

The number of poles increases with an increasing relative reaction strength *γ* in both the classic and non-classic case (Fig. 6). The number of poles has been calculated for a large range of the parameter *γ* (Fig. 7d) ranging from one single pole up to five poles. We believe that the random noise in the initial conditions is the reason for the fluctuations around the transitions between the number of poles. In addition, the relative reaction strength *γ* has no effect on the relative pole size (Fig. 7a) which varies around 25%. Again, these variations are almost certainly due to the random fluctuations in the initial data. Also, we observe that there is no clear relationship between *γ* and other quantitative measures such as the area of the pole relative to the surface area of the membrane (Fig. 7a), or the maximum and minimum concentration of active Cdc42 *u* (Fig. 7c). Similarly, this holds true for the classic case with respect to the time to polarisation (Fig. 7b) while this property seems to increase slightly with the relative reaction strength *γ* in the non-classic case. Thus, the two cases yield different predictions when it comes to the time to polarisation.

## Discussion

Cell polarisation is one of the most well-studied symmetry breaking events in biology using both experimental and theoretical approaches. Yet, it still remains largely unknown how complex, intertwined, and highly dynamic protein interactions control cell polarity. In this study, we constructed, analysed and verified a bulk-surface model of Cdc42-mediated cell polarisation. The analysis of the model resulted in a mathematical theorem showing the existence of multiple steady-states. In addition, a necessary condition for diffusion-driven instability was derived. Using a thorough numerical investigation of the parameter space, we have shown that the model can form patterns by means of both classic and non-classic Turing instability. Also, the simulations highlighted the connection between these two mechanisms where the non-classic case can be viewed as the classic case for equal diffusion rates of the membrane-bound species. Lastly, we validated the theoretical results by showing that both these mechanisms can sustain pattern formation. Using simulations, we propose that cell polarisation is mainly driven by a low value of the reaction strength parameter *γ*, that the size of the pole is determined by the relative diffusion *d* and that the effect of changing the kinetic parameters is quantitative rather than qualitative.

Within our bulk-surface formulation of the model, the membrane-bound reaction terms and the non-dimensionalisation are novel. The choice of the geometrical description that includes both the membrane and the cytosol in combination with adding the cytosolic GDI-bound form of Cdc42 to the model^21,26^ increases the level of realism. Previous models of the "wave-pinning” type^11,12,14,22^ have only focused on the two membrane-bound species and assumed mass conservation in the membrane, with one exception (ref. ^22^) that includes a cytosolic state but no extra reactions associated with it. We argue that from a biological perspective this is not entirely plausible as there is a fast-moving cytosolic state of Cdc42 that contributes to the transfer and dissociation reactions at the membrane. Furthermore, the introduced minimal reaction term *f* governing the activation–inactivation reactions is biologically motivated, where each term has a concrete meaning in terms of reaction rates. The non-dimensionalisation procedure implemented in the course of this work resulted in the derivation of biologically meaningful parameters such as *γ* corresponding to relative reaction strength and the activation parameter *c*_2_ corresponding to the membrane-bound reactions governing activation and inactivation.

Our exhaustive analysis of the parameter space shows that the relative activation rate *c*_2_ is higher than the relative dissociation rate from the membrane *c*_−1_ in the classic compared to the non-classic case where these two rates are more similar (Fig. 2). Biologically, the relative size between these parameters can be viewed as a kinetic "tug-of-war” between the two stable states of Cdc42, namely the cytosolic GDI-bound form and the membrane-bound GTP-bound form (Fig. 1c). Also, the investigation of the parameter space reveals that for certain activation rates *c*_2_, any value of the cytosolic flux to the membrane *c*_1_ allows for diffusion-driven instability in both cases (Fig. 2b). This suggests that a pattern will form independent of the cytosolic flux of GDI-bound Cdc42 to the membrane. Furthermore, a general conclusion drawn by studying the Turing parameter space is that in both the classic and non-classic case the activation rate *c*_2_ is larger in the former compared to the latter. In addition, our results suggest that the non-classic diffusion-driven instability is a special case of the classic one in the limit when *d* → 1 (Fig. 2).

Perhaps the most interesting result of this work is that cell polarisation can be modelled by both classic and the non-classic Turing patterns. This was demonstrated using numerical simulations, where we first showed that patterns can be formed through both mechanisms (Fig. 3). However, the time scales and dynamics of the two cases differ indicating that the mechanisms are different. The sensitivity of the final pattern in the two cases (Supplementary Fig. 4) with respect to variations in the kinetic parameters showed that the effect is quantitative rather than qualitative. More precisely, a mere change of parameters in the (*c*_−1_, *c*_2_)-plane does not alter the qualitative behaviour as a single pole is formed, however quantitative measures such as the time it takes to form the pole *τ*_final_ or the maximum concentration of active Cdc42 ${u}_{\max }$ are different for different kinetic parameters. In a similar investigation of the parameter space^27^, the existence of Turing patterns was investigated for 2-species and 3-species systems with a Hill function governing the interaction between the species. It was concluded that a large number of interaction topologies are capable of producing Turing patterns, but that they were not robust to parameter changes. Our results show that our model could be considered robust, since the parameter regions for which it produces Turing patterns is large. Nevertheless, the comparison between the two cases is not straightforward since the notion of robustness greatly depends on the parameter ranges in which the stability in investigated. This is also affected by, for example, the implementation of non-dimensionalisation which was not done in^27^.

In addition, we showed that the size of the pole, the time to polarisation and the maximum concentration ${u}_{\max }$ are influenced by the relative diffusion *d* (Fig. 4). This presents an opportunity for new experimental studies and for connecting the simulations of the bulk-surface models to data as a measure, however crude, of the size of the pole (for example as a percentage of the entire surface of the cell) that can be used to estimate the relative diffusion. This methodology for estimating the relative diffusion is consequential as it is currently not possible to differentiate between the three states of Cdc42 by using fluorescent markers and it is thereby not possible to estimate the relative diffusion *d* of the two membrane-bound species. Lastly, we showed that the key parameter determining the number of poles is the strength of the reaction term *γ* (Fig. 6). More precisely, one pole is formed for values of *γ* < 40 while numerous poles are formed for larger values, suggesting that the two classical parameters in reaction-diffusion models, *γ* and *d*, are consequential in the context of cell polarisation. These parameters govern the number of allowed wavenumbers^24^, where the smaller value of the parameters *γ* the fewer wave numbers contribute to the pattern formation and vice versa. This is in agreement with our simulations showing that the number of poles increases with *γ* (Fig. 7). In our model, this parameter is proportional to the surface of the cell (Table 1). Thus, as the size of the cell increases so does the number of poles which is in agreement with previous studies^28^. This indicates that cell polarisation, i.e. the formation of a single spot corresponding to a pole, can be achieved for both mechanisms as the formation of this particular pattern is dependent of the relative strengths of the reaction part *γ* and the diffusion part *d*. Thus, it is not qualitatively possible to rule out either the classic or the non-classic cases based on the patterns formed as both mechanisms can form a pole for low values of *γ*. However, it might be possible to quantitatively distinguish between the cases by studying the concentration profiles over time and comparing the time it takes for the patterns to be formed. Nevertheless, this poses experimental challenges as it is hard to connect high qualitative three-dimensional data based on microscopy with numerous images over time.

Understanding the underlying mechanisms of cell polarisation can shed light on many fundamental processes governing cell division and cell differentiation both during normal development and in the context of disease. Building, analysing and simulating Spatio-temporal models like the one proposed in this work can provide insight into mechanistic details as well as guide further experimental design. In the context of the budding event, both spatial and temporal aspects of bud emergence need to be considered. Here, we elucidate the complex interplay between the relative diffusion, the size of the pole, the time to polarisation and the concentration of active Cdc42 in the pole suggesting that perhaps cells have evolved multiple ways of maintaining these evolutionarily conserved phenomena.

## Methods

The representation of the parameter space (Fig. 2) has been generated using Matlab^29^. For the simulations, a combination of an adaptive solver based *Finite Differences* (*FD*) in time and the *Finite Element Method* (*FEM*) in space was implemented (Supplementary Text 2.2). As the numerical implementation solves a system of PDEs, a spatial discretisation is required in terms of a *mesh* over the domain Ω (Fig. 1a). For this purpose, the mesh was generated using the three-dimensional finite element mesh generator Gmsh^30^. For computational speed, we have implemented a non-uniform mesh with higher node-density close to the membrane and lower node-density in the cytosol as the former region requires more computational accuracy during the cell polarisation simulations than the latter. For the FD- and FEM-implementations, the computing platform FEniCS^31,32^ has been used. The visualisations (Fig. 3, Supplementary Figs. 4 and 6) have been constructed using the software ParaView^33,34^. For all the simulations, we have used a cytosolic diffusion coefficient of *D* = 10,000 and the spatially inhomogeneous initial conditions are perturbed around the steady states of the three states corresponding to the triplet (u^*^, v^*^, V^*^). More specifically, the steady state of interest is found numerically using a Newton solver which finds a solution to the equations *f*(u^*^, v^*^) = *q*_0_(u^*^, v^*^) = 0 where the functions *f* and *q*_0_ are presented in (8). The solution of interest is a pair (u^*^, v^*^) which satisfies the bound in (10) presented in Thm 3. Also, since the Newton solver is local in the sense that it finds a solution from a given start guess (*u*_0_, *v*_0_), this start guess is picked within the bound ${u}_{0}\in \left(\sqrt{{c}_{2}},\min \left\{{c}_{\max },m\right\}\right)$ based on Thm 3 where ${v}_{0}\leftarrow (({u}_{0})/({c}_{2}+{u}_{0}^{2}))$. Finally, the steady state of the cytosolic component is calculated by the equation V^*^ = *V*_0_ − *a*(u^*^ + v^*^) where the parameter *V*_0_ is set to *V*_0_ = 6 and the parameter *a* = ∣Γ∣/∣Ω∣ = (4*π*)/(4*π*/3) = 3 which is the quotient of the surface and the volume of the domain being the unit ball. To quantify polarisation properties, an empirical pole-recognition algorithm has been developed and implemented (Supplementary Text 2.3).

The simulations have been conducted on two computational clusters. The first is a Dell PowerEdge R730 with an Intel Xeon E5-2683 CPU and an NVIDIA Tesla K80 GPU. The second computational cluster is based on an Intel Xeon Platinum 8180 CPU. The total simulation time of all simulations presented in the paper was approximately one week.

### Reporting summary

Further information on research design is available in the Nature Research Reporting Summary linked to this article.

## Supplementary information

Supplementary file

reporting summary

## Data Availability

Data sharing not applicable to this article as no datasets were generated or analysed during the current study.

## Code availability

Scripts for simulating the Cdc42-mediated cell polarisation are avaliable at https://github.com/cvijoviclab/cdc42_ClassicalAndNonClassicalTuringConditions.
