# Supplementary material for: Cell polarisation in a bulk-surface model can be driven by both classic and non-classic Turing instability
Source: NPJ Syst Biol Appl. 2021 Feb 26;7:13. doi: 10.1038/s41540-021-00173-x (PMC7910310; doi:10.1038/s41540-021-00173-x)
Supplement: Supplementary file 1 — Supplementary file [file 41540_2021_173_MOESM1_ESM.pdf]

*Supplementary material to the article*

# Cell polarisation in a bulk-surface model can be driven by both classic and non-classic Turing instability

Johannes Borgqvist[1], Adam Malik[1], Carl Lundholm[1], Anders Logg[1], Philip Gerlee[1], Marija Cvijovic[1*]

**1** Department of Mathematical Sciences, Chalmers University of Technology and the University of Gothenburg, Gothenburg, Sweden.

Some scripts for simulating the Cdc42-mediated cell polarisation using the presented model and for generating some of the figures are available at:
`https://github.com/cvijoviclab/cdc42_ClassicalAndNonClassicalTuringConditions`.

*Corresponding Author: Marija Cvijovic, e-mail: `marija.cvijovic@chalmers.se`

# Contents


**Supplementary Text 1** **Analytical results** **1**
Supplementary Text 1.1 Bulk-Surface Activator-Inhibitor system . . . . 1
Supplementary Text 1.2 Non-dimensionalisation . . . . . . . . . . . . . . 2
Supplementary Text 1.3 Proof of Theorem 1 . . . . . . . . . . . . . . . . 6
Supplementary Text 1.4 Proof of Theorem 2 . . . . . . . . . . . . . . . . 10
Supplementary Text 1.4.1 Corollary 1 following from Theorem 2 . . . . . . 11
Supplementary Text 1.5 Diffusion driven instability . . . . . . . . . . . . 12
Supplementary Text 1.6 Proof of Theorem 3 . . . . . . . . . . . . . . . . 14
Supplementary Text 1.6.1 Existence of steady states . . . . . . . . . . . . 14
Supplementary Text 1.6.2 Steady states enabling diffusion-driven instability 15

**Supplementary Text 2** **Numerical implementations** **17**
Supplementary Text 2.1 Mapping of the parameter space . . . . . . . . . 18
Supplementary Text 2.2 Solving the RD-system numerically . . . . . . . 19
Supplementary Text 2.2.1 Setting up the problem . . . . . . . . . . . . . . 19
Supplementary Text 2.2.2 FEM-discretisation in space . . . . . . . . . . . 21
Supplementary Text 2.2.3 FD-discretisation in time . . . . . . . . . . . . . 23
Supplementary Text 2.3 Quantifying polarisation . . . . . . . . . . . . . 27

**Supplementary Text 3** **Additional results** **28**
Supplementary Text 3.1 Varying the kinetic parameters . . . . . . . . . 28
Supplementary Text 3.2 Effect of increasing diffusion with an absolute scale . . . . . . . . . . . . . . . . . . . . . . 30


# List of Figures


Supplementary figure 1 Steady states . . . . . . . . . . . . . . . . . . . . . . . 16
Supplementary figure 2 The spatial domain . . . . . . . . . . . . . . . . . . . . 20
Supplementary figure 3 Validation of the FEM-FD implementation . . . . . . . . . 26
Supplementary figure 4 Final patterns for parameters in the $(\mathbf{c_{-1}}, \mathbf{c_2})$-plane . . . . 28
Supplementary figure 5 Final patterns for parameters in the $(\mathbf{c_1}, \mathbf{c_2})$-plane . . . . . 29
Supplementary figure 6 Final patterns for increasing diffusion . . . . . . . . . . . 30

# Supplementary Text 1 Analytical results

The analytical results consists of three parts: (1) A detailed description of as well as a motivation behind the bulk-surface activator-inhibitor system; (2) the details of the non-dimensionalisation procedure; (3) the proofs of all theorems (i.e. Theorem 1, Theorem 2 and Theorem 3) in the article.

## Supplementary Text 1.1 Description of the bulk-surface Activator-Inhibitor system

### Influx of Inactive Cdc42 from the cytosol

The influx of inactive Cdc42 in the vicinity of the membrane is determined by the concentration of GDI-bound Cdc42 in the cytosol (Fig 1C in the article). The influx of the inactive GDP-bound form of Cdc42 is proportional to $G$ where the rate of the reaction is determined by the rate constant $k_1$ with units "$\text{m}^3/\text{min}$". We also assume that there exists a saturation level of active and inactive form of Cdc42 on the membrane, denoted by $k_{\text{max}}$. These two assumptions result in the reaction rate given by:

$$\text{Influx rate} = k_1 \cdot G \cdot (k_{\text{max}} - (A + I)) .$$

### Dissociation of Inactive Cdc42 from the membrane

For the dissociation of GDP-bound Cdc42 from the membrane, we assume a first order reaction with rate constant $k_{-1}$ with units "m/min", which results in:

$$\text{Dissociation rate} = k_{-1} \cdot I.$$

### Activation of Inactive Cdc42

The activation of inactive Cdc42 which occurs on the membrane corresponds to the conversion of GDP-bound Cdc42 to the GTP-bound form. The reaction rate is assumed to be proportional to the concentration of active GDP-bound form of Cdc42, and the rate constant $k_2$ with units "$\text{min}^{-1}$" is assumed to be proportional to the concentration of GEFs, which we take to be constant during the time scale on which polarisation occurs. This results in a first order reaction term given by:

$$\text{Activation rate} = k_2 \cdot I.$$

### Inactivation of Active Cdc42

The inactivation of active Cdc42 corresponds to the conversion of GTP-bound Cdc42 to the GDP-bound form. Similar to the activation rate, it is also assumed to be a first order reaction, where the rate coefficient $k_{-2}$ with units "$\text{min}^{-1}$" is assumed to be proportional to the concentration of GAPs, taken to be constant on the time scale of polarisation. This results in the following reaction rate:

$$\text{Inactivation rate} = k_{-2} \cdot A.$$

### Activation of Cdc42 through a positive feedback loop

The feedback loop consists of the binding of active Cdc42 to PAKs which forms a complex which can further bind to various scaffolds. Together, this sequence of events forms a structure which can bind more GEFs and thereby enhance the activation process. During the time scale that polarisation occurs, we assume that both the concentration of PAKs and GEFs are constant,

and that the feedback loop that recruits GEF is nonlinear. Such a feedback mechanism has been proposed previously (Supplementary Reference [13]) and takes the form:

$$\text{Feedback activation rate} = k_3 \cdot A^2 \cdot I.$$

where the product "$k_3A$" can be interpreted as a second order reaction rate constant which requires both $A$ and $I$ to occur. The unit of $k_3$ is "m$^4$/min".

## Supplementary Text 1.2 Non-dimensionalisation of the model

The aim of the non-dimensionalisation is to render the following model:

$$\begin{aligned}
\frac{\partial A}{\partial t} &= \underbrace{k_2 I - k_{-2}A + k_3 A^2 I}_{=F(A,I)} + D_A \Delta A \\
&= F(A,I) + D_A \Delta A \\
\\
\frac{\partial I}{\partial t} &= \underbrace{-F(A,I) + Q(A,I,G)}_{=G(A,I)} + D_I \Delta I \\
&= G(A,I) + D_I \Delta I \\
\frac{\partial G}{\partial t} &= D_G \Delta G \\
-D\left((\nabla G)^T \cdot \mathbf{n}\right) &= k_1 V(k_{\max} - (A+I)) - k_1 I \\
&= Q(A,I,G)
\end{aligned} \tag{11}$$

dimensionless. To this end, we follow the standard procedure in the context of dynamical models in mathematical biology which entails that we non-dimensionalise the model in order to reduce the number of parameters. Our choice of non-dimensional parameters are similar to the ones in the classical Schnackenberg model (Supplementary Reference [35]). Firstly, we introduce the following dimensionless states

$$\begin{aligned}
u &= A \cdot \sqrt{\frac{k_3}{k_{-2}}}, \\
v &= I \cdot \sqrt{\frac{k_3}{k_{-2}}}, \\
V &= G \cdot \frac{1}{R}\sqrt{\frac{k_3}{k_{-2}}},
\end{aligned} \tag{12}$$

and the following dimensionless variables

$$\begin{aligned}
\tau &= \frac{D_A t}{R^2}, \\
\\
\mathbf{x} &\leftarrow \frac{1}{R}\mathbf{x}.
\end{aligned} \tag{13}$$

Note, that after the introduction of the spatial variable $\mathbf{x}$, the domain $\Omega$ is transformed to the *unit ball* $\Omega := \left\{\mathbf{x} \in \mathbb{R}^3 : \|\mathbf{x}\|_2^2 < 1\right\}$ where the membrane described by $\Gamma := \left\{\mathbf{x} \in \mathbb{R}^3 : \|\mathbf{x}\|_2^2 = 1\right\}$ corresponds to the *unit sphere*.

By substituting the proposed scalings of the states in Supplementary Equation (12) and the variables in Supplementary Equation (13), we will derive the non-dimensional version of the model in Supplementary Equation (11). We start with the left hand side and the time derivatives in order to obtain:

$$\begin{aligned} A &= \sqrt{\frac{k_{-2}}{k_3}}u, \\ I &= \sqrt{\frac{k_{-2}}{k_3}}v, \\ G &= \frac{1}{R}\sqrt{\frac{k_{-2}}{k_3}}V, \end{aligned}$$

where the time variable is the following:

$$t = \frac{R^2}{D_A}\tau.$$

These expressions result in the following left hand sides of the PDEs in Supplementary Equation (11)

$$\begin{aligned} \frac{\partial A}{\partial t} &= \left(\frac{D_A}{R^2}\sqrt{\frac{k_{-2}}{k_3}}\right)\frac{\partial u}{\partial \tau} \\ \frac{\partial I}{\partial t} &= \left(\frac{D_A}{R^2}\sqrt{\frac{k_{-2}}{k_3}}\right)\frac{\partial v}{\partial \tau} \\ \frac{\partial G}{\partial t} &= \left(\frac{D_A}{R^3}\sqrt{\frac{k_{-2}}{k_3}}\right)\frac{\partial V}{\partial \tau} \end{aligned}$$

and thus the aim is to factor out "$\left(\frac{D_A}{R^2}\sqrt{\frac{k_{-2}}{k_3}}\right)$" from the remaining terms in Supplementary Equation (11): $F(A, I)$, $G(A, I)$, and the diffusion terms. For the diffusion terms, in the one-dimensional case[1], i.e. $x \in [0, L]$, it follows that the following holds

$$\begin{aligned} D_A \Delta A &= D_A \frac{\partial^2}{\partial x^2}A \\ &\quad \{\text{Use the non-dimensional assignment for the spatial variable } x \text{ (Supplementary Equation (13))}\} \\ &= \frac{D_A}{L^2}\frac{\partial^2}{\partial x^2}A = \frac{D_A}{L^2}\Delta A \\ &\quad \{\text{Substitute the non-dimensional state } u \text{ in place of } A \text{ (Supplementary Equation (12))}\} \\ &= \left(\frac{D_A}{L^2}\sqrt{\frac{k_{-2}}{k_3}}\right)\Delta u. \end{aligned}$$

[1]The spherical case is analogous.

Analogously, in the spherical case with the spherical Laplace operator the factor $L^2$ in the denominator in the one-dimensional case is replaced by the square of the radius $R^2$ which is summarised as follows

$$D_A \Delta A = \left( \frac{D_A}{R^2} \sqrt{\frac{k_{-2}}{k_3}} \right) \Delta u.$$

Similarly, for $I$

$$D_I \Delta I = \left( \frac{D_A}{R^2} \sqrt{\frac{k_{-2}}{k_3}} \right) \frac{D_I}{D_A} \Delta v$$

and for $G$ the following holds

$$D_G \Delta G = \left( \frac{D_A}{R^3} \sqrt{\frac{k_{-2}}{k_3}} \right) \frac{D_G}{D_A} \Delta V.$$

For the reaction term $F(A, I)$, we have

$$\begin{aligned}
F(A, I) &= k_2 I - k_{-2} A + k_3 A^2 I \\
&= \left( \frac{D_A}{R^2} \sqrt{\frac{k_{-2}}{k_3}} \right) \left( \frac{R^2}{D_A} \sqrt{\frac{k_3}{k_{-2}}} \right) \left( k_2 I - k_{-2} A + k_3 A^2 I \right) \\
&= \left( \frac{D_A}{R^2} \sqrt{\frac{k_{-2}}{k_3}} \right) \left( \frac{R^2}{D_A} \right) \left( k_2 \underbrace{\left[ \sqrt{\frac{k_3}{k_{-2}}} I \right]}_{=v} - k_{-2} \underbrace{\left[ \sqrt{\frac{k_3}{k_{-2}}} A \right]}_{=u} + k_3 \underbrace{A^2}_{=\left(\frac{k_{-2}}{k_3}\right) u^2} \underbrace{\left[ \sqrt{\frac{k_3}{k_{-2}}} I \right]}_{=v} \right) \\
&= \left( \frac{D_A}{R^2} \sqrt{\frac{k_{-2}}{k_3}} \right) \left( \frac{R^2}{D_A} \right) \left( k_2 v - k_{-2} u + k_3 \left( \frac{k_{-2}}{k_3} \right) u^2 v \right) \\
&= \left( \frac{D_A}{R^2} \sqrt{\frac{k_{-2}}{k_3}} \right) \left( \frac{R^2 k_{-2}}{D_A} \right) \left( \frac{k_2}{k_{-2}} v - u + u^2 v \right)
\end{aligned}$$

which is summarised as follows

$$F(A, I) = \left( \frac{D_A}{R^2} \sqrt{\frac{k_{-2}}{k_3}} \right) \left( \frac{R^2 k_{-2}}{D_A} \right) \left( \frac{k_2}{k_{-2}} v - u + u^2 v \right) = \left( \frac{D_A}{R^2} \sqrt{\frac{k_{-2}}{k_3}} \right) \left( \frac{R^2 k_{-2}}{D_A} \right) f(u, v).$$

Lastly, we would like to factor out $\left( \frac{D_A}{R^2} \sqrt{\frac{k_{-2}}{k_3}} \right) \left( \frac{R^2 k_{-2}}{D_A} \right)$ from the above expression of $F(A, I)$ from $G(A, I)$. However, as $G(A, I) = -F(A, I) + Q(A, I, G)$ it suffices to look at the the transfer function $Q(A, I, G)$. In a similar fashion, we obtain

$$
\begin{aligned}
Q(A,I,G) &= k_1 G(k_{\max} - (A+I)) - k_{-1} I \\
&= \left[\left(\frac{D_A}{R^2}\sqrt{\frac{k_{-2}}{k_3}}\right)\left(\frac{R^2 k_{-2}}{D_A}\right)\right]\left(\frac{1}{k_{-2}}\sqrt{\frac{k_3}{k_{-2}}}\right)\{k_1 G(k_{\max} - (A+I)) - k_{-1} I\} \\
&= \left[\left(\frac{D_A}{R^2}\sqrt{\frac{k_{-2}}{k_3}}\right)\left(\frac{R^2 k_{-2}}{D_A}\right)\right]\left\{\frac{k_1}{k_{-2}}\left[\underbrace{\sqrt{\frac{k_3}{k_{-2}}}G}_{=V}\right](k_{\max} - (A+I)) - \frac{k_{-1}}{k_{-2}}\left[\underbrace{\sqrt{\frac{k_3}{k_{-2}}}I}_{=v}\right]\right\} \\
&= \left[\left(\frac{D_A}{R^2}\sqrt{\frac{k_{-2}}{k_3}}\right)\left(\frac{R^2 k_{-2}}{D_A}\right)\right]\left\{\frac{k_1}{k_{-2}}V(k_{\max} - (A+I)) - \frac{k_{-1}}{k_{-2}}v\right\} \\
&= \left[\left(\frac{D_A}{R^2}\sqrt{\frac{k_{-2}}{k_3}}\right)\left(\frac{R^2 k_{-2}}{D_A}\right)\right]\left\{\frac{k_1}{k_{-2}}\sqrt{\frac{k_{-2}}{k_3}}V\left(k_{\max}\sqrt{\frac{k_3}{k_{-2}}} - \left[\underbrace{\sqrt{\frac{k_3}{k_{-2}}}(A+I)}_{=(u+v)}\right]\right) - \frac{k_{-1}}{k_{-2}}v\right\} \\
&= \left[\left(\frac{D_A}{R^2}\sqrt{\frac{k_{-2}}{k_3}}\right)\left(\frac{R^2 k_{-2}}{D_A}\right)\right]\left\{\frac{k_1}{k_{-2}}\sqrt{\frac{k_{-2}}{k_3}}V\left(k_{\max}\sqrt{\frac{k_3}{k_{-2}}} - (u+v)\right) - \frac{k_{-1}}{k_{-2}}v\right\}
\end{aligned}
$$

which yields the following result

$$
\begin{aligned}
Q(A,I,G) &= \left[\left(\frac{D_A}{R^2}\sqrt{\frac{k_{-2}}{k_3}}\right)\left(\frac{R^2 k_{-2}}{D_A}\right)\right]\left\{\frac{k_1}{k_{-2}}\sqrt{\frac{k_{-2}}{k_3}}V\left(k_{\max}\sqrt{\frac{k_3}{k_{-2}}} - (u+v)\right) - \frac{k_{-1}}{k_{-2}}v\right\} \\
&= \left[\left(\frac{D_A}{R^2}\sqrt{\frac{k_{-2}}{k_3}}\right)\left(\frac{R^2 k_{-2}}{D_A}\right)\right] q(u,v,V).
\end{aligned}
$$

Now, summarising all these terms yields the following

$$
\begin{aligned}
\left(\frac{D_A}{R^2}\sqrt{\frac{k_{-2}}{k_3}}\right)\frac{\partial u}{\partial \tau} &= \left(\frac{D_A}{R^2}\sqrt{\frac{k_{-2}}{k_3}}\right)\left\{\left(\frac{R^2 k_{-2}}{D_A}\right)\left(\frac{k_2}{k_{-2}}v - u + u^2 v\right) + \Delta u\right\} \\
\left(\frac{D_A}{R^2}\sqrt{\frac{k_{-2}}{k_3}}\right)\frac{\partial v}{\partial \tau} &= \left(\frac{D_A}{R^2}\sqrt{\frac{k_{-2}}{k_3}}\right)\left\{\left(\frac{R^2 k_{-2}}{D_A}\right)\left[-\left(\frac{k_2}{k_{-2}}v - u + u^2 v\right) + \right.\right. \\
&\left.\left.\left\{\frac{k_1}{k_{-2}}\sqrt{\frac{k_{-2}}{k_3}}V\left(k_{\max}\sqrt{\frac{k_3}{k_{-2}}} - (u+v)\right) - \frac{k_{-1}}{k_{-2}}v\right\}\right] + \frac{D_I}{D_A}\Delta v\right\} \\
\left(\frac{D_A}{R^3}\sqrt{\frac{k_{-2}}{k_3}}\right)\frac{\partial V}{\partial \tau} &= \left(\frac{D_A}{R^3}\sqrt{\frac{k_{-2}}{k_3}}\right)\frac{D_G}{D_A}\Delta G \\
\left(\frac{D_A}{R^2}\sqrt{\frac{k_{-2}}{k_3}}\right)\left\{-\frac{D_G}{D_A}\left((\nabla V)^T \cdot \mathbf{n}\right)\right\} &= \left[\left(\frac{D_A}{R^2}\sqrt{\frac{k_{-2}}{k_3}}\right)\left(\frac{R^2 k_{-2}}{D_A}\right)\right] \\
&\left\{\frac{k_1}{k_{-2}}\sqrt{\frac{k_{-2}}{k_3}}V\left(k_{\max}\sqrt{\frac{k_3}{k_{-2}}} - (u+v)\right) - \frac{k_{-1}}{k_{-2}}v\right\}
\end{aligned}
$$

and cancelling the common factor in both sides results in the following equation

$$\begin{aligned}
\frac{\partial u}{\partial \tau} &= \left(\frac{R^2 k_{-2}}{D_A}\right)\left(\frac{k_2}{k_{-2}}v - u + u^2 v\right) + \Delta u\\
\frac{\partial v}{\partial \tau} &= \left(\frac{R^2 k_{-2}}{D_A}\right)\left[-\left(\frac{k_2}{k_{-2}}v - u + u^2 v\right) + \left\{\frac{k_1}{k_{-2}}\sqrt{\frac{k_{-2}}{k_3}}V\left(k_{\max}\sqrt{\frac{k_3}{k_{-2}}} - (u+v)\right) - \frac{k_{-1}}{k_{-2}}v\right\}\right] + \frac{D_I}{D_A}\Delta v\\
\frac{\partial V}{\partial \tau} &= \frac{D_G}{D_A}\Delta V\\
\left\{-\frac{D_G}{D_A}\left((\nabla V)^T \cdot \mathbf{n}\right)\right\} &= \left(\frac{R^2 k_{-2}}{D_A}\right)\left\{\frac{k_1}{k_{-2}}\sqrt{\frac{k_{-2}}{k_3}}V\left(k_{\max}\sqrt{\frac{k_3}{k_{-2}}} - (u+v)\right) - \frac{k_{-1}}{k_{-2}}v\right\}.
\end{aligned}$$

The introduction of the following parameters

$$c_1 = \frac{k_1}{k_{-2}}\sqrt{\frac{k_{-2}}{k_3}},\ \ c_{\max} = k_{\max}\sqrt{\frac{k_3}{k_{-2}}},\ \ c_{-1} = \frac{k_{-1}}{k_{-2}},\ \ c_2 = \frac{k_2}{k_{-2}},\ \ \gamma = \frac{R^2 k_{-2}}{D_A},\ \ D = \frac{D_G}{D_A},\ \text{ and }\ d = \frac{D_I}{D_A}$$

results in the following equations

$$\begin{aligned}
\frac{\partial u}{\partial \tau} &= \gamma\left(c_2 v - u + u^2 v\right) + \Delta u\\
\frac{\partial v}{\partial \tau} &= \gamma\left(-\left(c_2 v - u + u^2 v\right) + \left(c_1 V(c_{\max} - (u+v)) - c_{-1}v\right)\right) + d\Delta v\\
\frac{\partial V}{\partial \tau} &= D\Delta V\\
-D\left((\nabla V)^T \cdot \mathbf{n}\right) &= \gamma\left[c_1 V(c_{\max} - (u+v)) - c_{-1}v\right]
\end{aligned}$$

which is the desired result.

□

## Supplementary Text 1.3 Proof of Theorem 1

The proof is given by re-writing the PDEs on integral form using Duhamel's principle and then use Banach's contraction theorem on the corresponding mappings resulting from these integral forms. Similar existence proofs are well-studied for other RD models, see for example Supplementary Reference [22], but in this case the proof must be adapted for the unit sphere.

To this end, we equip the manifold $\Gamma$ with a measure $\mathrm{d}\omega$ defined by

$$\int_\Gamma \mathrm{d}\omega(\mathbf{x}) = \int_0^\pi \int_0^{2\pi} \sin(\phi)\ \mathrm{d}\phi \mathrm{d}\theta,\ \ \mathbf{x} \in \Gamma$$

which implies that standard spherical coordinates corresponding to the angle $\theta \in [0, 2\pi]$ and the angle $\phi \in [0, \pi]$ are implemented. Also, we define the corresponding $L_2$-inner product and norm as follows

$$\langle f, g\rangle_{\mathcal{L}_2(\Gamma)} = \int_\Gamma f(\mathbf{x})g(\mathbf{x})\mathrm{d}\omega(\mathbf{x}) \Longrightarrow \langle f, f\rangle_{\mathcal{L}_2(\Gamma)} = \|f\|^2_{\mathcal{L}_2(\Gamma)}$$

where we define the related $\mathcal{L}_2$-space as follows

$$\mathcal{L}_2(\Gamma) := \left\{\text{Functions } f : \|f\|_{\mathcal{L}_2(\Gamma)} < \infty\right\}.$$

Using this norm, we can also define the $\mathcal{H}^1(\Gamma)$-norm according to

$$\|f\|^2_{\mathcal{H}^1(\Gamma)} := \|f\|^2_{\mathcal{L}_2(\Gamma)} + \|\nabla_\Gamma f\|^2_{\mathcal{L}_2(\Gamma)},$$

where $\nabla_\Gamma$ is the surface gradient in a weak sense with respect to $\Gamma$. This so called $\mathcal{H}^1$-norm requires that both the function value and its derivative do not "blow up". Now, we can define our Sobolev space of interest, denoted by $\mathcal{H}^1(\Gamma)$, as follows

$$\mathcal{H}^1(\Gamma) := \left\{f \in \mathcal{L}_2(\Gamma) : \|f\|_{\mathcal{H}^1(\Gamma)} < \infty\right\}.$$

The notation $|f|_{\mathcal{H}^1(\Gamma)}$ is sometimes used for bounding only the derivative, i.e.

$$|f|^2_{\mathcal{H}^1(\Gamma)} = \|\nabla_\Gamma f\|^2_{\mathcal{L}_2(\Gamma)}.$$

Now, an *orthonormal* (*ON*)-basis for the Hilbert space of $L_2$-functions on the sphere with the $L_2$-scalar product defined above is the *Legendre polynomials* (Supplementary Reference [11]) which we will denote by $P_n$[2]. Based on this, we can define two so called *solution operators* (Supplementary Reference [27]) $E_1(\tau)$ and $E_2(\tau)$ respectively acting on a function $v$ as follows

$$(E_1(\tau)v)(\mathbf{x}) = \sum_{n=0}^{\infty} e^{-\lambda_n \tau}\langle v, P_n\rangle_{\mathcal{L}_2(\Gamma)} P_n(\mathbf{x}),\ \mathbf{x} \in \Gamma,$$
$$(E_2(\tau)v)(\mathbf{x}) = \sum_{n=0}^{\infty} e^{-\lambda_n d\, \tau}\langle v, P_n\rangle_{\mathcal{L}_2(\Gamma)} P_n(\mathbf{x}),\ \mathbf{x} \in \Gamma,$$

where the solution operator corresponds to calculating the Fourier series a given function $v \in \mathcal{L}_2(\Gamma)$. Here, the parameters $\lambda_n$ correspond to the eigenvalues of the Laplace-Beltrami operator, and for an asymptotic expansion of the various eigenvalues $\lambda_n$, see Supplementary Reference [18]. Moreover, by using the inequality "$xe^{-x} \leq C$" for the exponential function it is possible to show the following bounds

$$\|E(\tau)v\|_{\mathcal{H}^1(\Gamma)} \leq C\,\|v\|_{\mathcal{H}^1(\Gamma)},$$
$$\|E(\tau)v\|_{\mathcal{H}^1(\Gamma)} \leq \hat{C}\tau^{-1/2}\,\|v\|_{\mathcal{L}^2(\Gamma)},$$

for a general solution operator $E$ (i.e. the above bounds hold both for $E_1$ and $E_2$). The first of these bounds follow from the fact that

$$|E(\tau)v|_{\mathcal{H}^1(\Gamma)} \leq \sqrt{\left(\sum_{n=0}^{\infty} e^{-2\lambda_n \tau}\lambda_n\langle v, P_n\rangle^2_{\mathcal{L}_2(\Gamma)}\right)} \leq C|v|_{\mathcal{H}^1(\Gamma)}.$$

It is in the derivation of the second of these inequalities that the previously mentioned inequality "$xe^{-x} \leq C$" can be used as follows

$$\|E(\tau)v\|_{\mathcal{H}^1(\Gamma)} = \sqrt{\left(\sum_{n=0}^{\infty} e^{-2\lambda_n \tau}\lambda_n\langle v, P_n\rangle^2_{\mathcal{L}_2(\Gamma)}\right)} \leq C\tau^{-1/2}\|v\|_{\mathcal{L}^2(\Gamma)}.$$

[2]A closely connected concept is that of fundamental solutions which are also expressed in terms of these polynomials. For a specific example of what the fundamental solutions to the heat equation on the unit sphere look like in the case of rotational invariance, see Supplementary Reference [43].

Given these solution operators, for a time $\tau \in [0, \tau_{\max}]$, the solution components $u(\tau), v(\tau) \in \mathcal{H}^1(\Gamma)$ of Supplementary Equation (18) can be written on integral form according to Duhamel's principle:

$$u(\tau) = E_1(\tau)u_0 + \int_0^\tau E_1(\tau - s)f(u(s), v(s))\mathrm{d}s,$$
$$v(\tau) = E_2(\tau)v_0 + \int_0^\tau E_2(\tau - s)(-f(u(s), v(s)) + q(u(s), v(s), V(s)))\mathrm{d}s.$$

In fact, we can view these integral forms as operators $T_1$ and $T_2$ defined according to

$$T_1(u, v)(\tau) := E_1(\tau)u_0 + \int_0^\tau E_1(\tau - s)f(u(s), v(s))\mathrm{d}s, \tag{14}$$
$$T_2(u, v)(\tau) := E_2(\tau)v_0 + \int_0^\tau E_2(\tau - s)(-f(u(s), v(s)) + q(u(s), v(s), V(s)))\mathrm{d}s, \tag{15}$$

which strongly motivates us to consider the system on vector form. To this end, we denote the states on vector form as follows

$$\mathbf{u} = (u, v).$$

We define the operator $\mathbf{T}$ by, for a time $\tau \in [0, \tau_{\max}]$

$$\mathbf{T}(\mathbf{u})(\tau) := (T_1(\mathbf{u})(\tau), T_2(\mathbf{u})(\tau)) = (T_1(u, v)(\tau), T_2(u, v)(\tau)).$$

We note that for a time $\tau \in [0, \tau_{\max}]$, $\mathbf{u}(\tau) = (u(\tau), v(\tau)) \in \mathcal{H}^1(\Gamma) \times \mathcal{H}^1(\Gamma)$. We equip this product space with the norm $\|\cdot\|_{\mathcal{H}^1\times\mathcal{H}^1}$ defined by

$$\|\mathbf{u}(\tau)\|_{\mathcal{H}^1\times\mathcal{H}^1} := \|u(\tau)\|_{\mathcal{H}^1(\Gamma)} + \|v(\tau)\|_{\mathcal{H}^1(\Gamma)}.$$

Let $X = \mathcal{C}([0, \tau_{\max}]; \mathcal{H}^1(\Gamma) \times \mathcal{H}^1(\Gamma))$ denote the Banach space of functions that are continuous in time on the interval $[0, \tau_{\max}]$ and for a fixed $\tau$-value take values in $\mathcal{H}^1(\Gamma) \times \mathcal{H}^1(\Gamma)$ (a so called Bochner space). Here, this space is equipped with the following norm

$$\|\mathbf{u}\|_X := \max_{\tau\in[0,\tau_{\max}]} \|\mathbf{u}(\tau)\|_{\mathcal{H}^1\times\mathcal{H}^1} = \max_{\tau\in[0,\tau_{\max}]} \left\{ \|u(\tau)\|_{\mathcal{H}^1(\Gamma)} + \|v(\tau)\|_{\mathcal{H}^1(\Gamma)} \right\}.$$

We now define a closed subspace of this Banach space by the ball $\mathcal{B}$ as follows

$$\mathcal{B} := \{\mathbf{u} \in X : \|\mathbf{u}\|_X \leq R\}$$

and furthermore we have the following bounds for the reaction functions $f, q$

$$\|f(u_1, v_1) - f(u_2, v_2)\|_{\mathcal{L}_2(\Gamma)} \leq \tilde{C}_1 \left(\|u_1 - u_2\|_{\mathcal{H}^1(\Gamma)} + \|v_1 - v_2\|_{\mathcal{H}^1(\Gamma)}\right) \leq C_1(R),$$
$$\|q(u_1, v_1) - q(u_2, v_2)\|_{\mathcal{L}_2(\Gamma)} \leq \tilde{C}_2 \left(\|u_1 - u_2\|_{\mathcal{H}^1(\Gamma)} + \|v_1 - v_2\|_{\mathcal{H}^1(\Gamma)}\right) \leq C_2(R),$$

due to the fact that both these functions are continuous with continuous derivatives, i.e. $f, q \in \mathcal{C}^1(\mathbb{R}^2)$. In addition, since $f(0, 0) = 0$ and since $q(0, 0) = c_1 c_{\max} V_0$ is bounded, we also have the following bounds

$$\|f(u,v)\|_{\mathcal{L}_2(\Gamma)} \leq \tilde{C}_1 \left(\|u\|_{\mathcal{H}^1(\Gamma)} + \|v\|_{\mathcal{H}^1(\Gamma)}\right) \leq C_1(R),$$
$$\|q(u,v) - c_1 c_{\max} V_0\|_{\mathcal{L}_2(\Gamma)} \leq \tilde{C}_2 \left(\|u\|_{\mathcal{H}^1(\Gamma)} + \|v\|_{\mathcal{H}^1(\Gamma)}\right) \leq C_2(R)$$

for some constants $C_1, C_2$ depending on the radius of the ball $\mathcal{B}$. Moreover, since $q \in \mathcal{C}^1(\mathbb{R}^2)$ it follows that we can find a constant $C_2(R)$ such that $\|q(u,v)\|_{\mathcal{L}_2(\Gamma)} \leq C_2(R)$.

Note here, that since the initial conditions $u_0, v_0$ are chosen in the region $\mathcal{A}$ the upper bound on these initial conditions are known, more precisely the following holds

$$\|u_0\|^2_{\mathcal{L}_2(\Gamma)} \leq \min\{c_{\max}, m\}^2 |\Gamma| = 4\pi \min\{c_{\max}, m\}^2,$$
$$\|v_0\|^2_{\mathcal{L}_2(\Gamma)} \leq \min\{c_{\max}, m\}^2 |\Gamma| = 4\pi \min\{c_{\max}, m\}^2.$$

Also, it assumed in the theorem that the derivatives are bounded, so let us denote the upper bound of the derivatives by the constant $M$ implying that the following holds

$$\|\nabla u_0\|^2_{\mathcal{L}_2(\Gamma)} \leq M \text{ and } \|\nabla v_0\|^2_{\mathcal{L}_2(\Gamma)} \leq M$$

and combining these bounds yield

$$\|u_0\|_{\mathcal{H}^1(\Gamma)} \leq \sqrt{(4\pi \min\{c_{\max}, m\}^2 + M)} \text{ and } \|v_0\|_{\mathcal{H}^1(\Gamma)} \leq \sqrt{(4\pi \min\{c_{\max}, m\}^2 + M)}.$$

Next, we define a fixed point $\mathbf{u}^\star \in \mathcal{B}$ as follows

$$\mathbf{T}(\mathbf{u}^\star) = \mathbf{u}^\star$$

and the existence of a *unique* such point is guaranteed by *Banach's fixed point theorem* (Supplementary Reference [10]) if the following two conditions are satisfied:

1. The operator maps the closed subspace to the closed subspace, i.e. $\mathbf{T} : \mathcal{B} \to \mathcal{B}$. In other words, the image of the operator lies in the closed subspace of the Banach space, i.e.
$$\mathbf{T}(\mathbf{u}) \in \mathcal{B}, \quad \forall \mathbf{u} \in \mathcal{B},$$
2. The operator is a contraction, i.e.
$$\exists \alpha \in [0,1) : \|\mathbf{T}(\mathbf{u}_2) - \mathbf{T}(\mathbf{u}_1)\|_X \leq \alpha \|\mathbf{u}_2 - \mathbf{u}_1\|_X, \quad \forall \mathbf{u}_1, \mathbf{u}_2 \in \mathcal{B}.$$

Regarding the first condition, we have

$$\begin{aligned}
\|\mathbf{T}(\mathbf{u})\|_X &= \max_{\tau \in [0,\tau_{\max}]} \|\mathbf{T}(\mathbf{u})(\tau)\|_{\mathcal{H}^1 \times \mathcal{H}^1} = \max_{\tau \in [0,\tau_{\max}]} \left\{ \|T_1(u,v)(\tau)\|_{\mathcal{H}^1(\Gamma)} + \|T_2(u,v)(\tau)\|_{\mathcal{H}^1(\Gamma)} \right\} \\
&\leq \max_{\tau \in [0,\tau_{\max}]} \|T_1(u,v)\|_{\mathcal{H}^1(\Gamma)} + \max_{\tau \in [0,\tau_{\max}]} \|T_2(u,v)\|_{\mathcal{H}^1(\Gamma)} \\
&\leq \max_{\tau \in [0,\tau_{\max}]} \|E_1(\tau)u_0\|_{\mathcal{H}^1(\Gamma)} + \max_{\tau \in [0,\tau_{\max}]} \int_0^\tau \|E_1(\tau - s)f\|_{\mathcal{H}^1(\Gamma)} \, \mathrm{d}s \\
&+ \max_{\tau \in [0,\tau_{\max}]} \|E_2(\tau)v_0\|_{\mathcal{H}^1(\Gamma)} + \max_{\tau \in [0,\tau_{\max}]} \int_0^\tau \|E_2(\tau - s)(-f + q)\|_{\mathcal{H}^1(\Gamma)} \, \mathrm{d}s \\
&\leq \|u_0\|_{\mathcal{H}^1(\Gamma)} + \max_{\tau \in [0,\tau_{\max}]} \int_0^\tau \hat{C}_1 (\tau - s)^{-1/2} \|f\|_{\mathcal{L}_2(\Gamma)} \, \mathrm{d}s \\
&+ \|v_0\|_{\mathcal{H}^1(\Gamma)} + \max_{\tau \in [0,\tau_{\max}]} \int_0^\tau \hat{C}_2 (\tau - s)^{-1/2} (\|f\|_{\mathcal{L}_2(\Gamma)} + \|q\|_{\mathcal{L}_2(\Gamma)}) \mathrm{d}s \\
&\leq 2\sqrt{(4\pi \min\{c_{\max}, m\}^2 + M)} + ((\tilde{C}_1 + \tilde{C}_2) C_1(R) + C_2(R)) \tau_{\max}^{1/2}.
\end{aligned}$$

Now, if we choose

$$R = 4\sqrt{(4\pi \min\{c_{\max}, m\}^2 + M)}$$

and by choosing $\tau_{\max}$ sufficiently small so that

$$((\tilde{C}_1 + \tilde{C}_2)C_1(R) + C_2(R))\tau_{\max}^{1/2} \leq \frac{1}{2}R$$

we have that $\|\mathbf{T}(\mathbf{u})\|_X \leq R$ and hence $\mathbf{T} : \mathcal{B} \to \mathcal{B}$.

In order to prove the second condition, we take $\mathbf{u}_1 = (u_1, v_1)$ and $\mathbf{u}_2 = (u_2, v_2)$ such that $\mathbf{u}_1, \mathbf{u}_2 \in \mathcal{B}$. It follows that

$$\begin{aligned}
\|\mathbf{T}(\mathbf{u}_1) - \mathbf{T}(\mathbf{u}_2)\|_X \leq & \max_{\tau\in[0,\tau_{\max}]} \int_0^\tau \|E_1(\tau - s)(f(u_1, v_1) - f(u_2, v_2))\|_{\mathcal{H}^1(\Gamma)} \,\mathrm{d}s + \\
& \max_{\tau\in[0,\tau_{\max}]} \int_0^\tau \|E_2(\tau - s)(-(f(u_1, v_1) - f(u_2, v_2)) + (q(u_1, v_1) - q(u_2, v_2)))\|_{\mathcal{H}^1(\Gamma)} \,\mathrm{d}s \\
& \leq \max_{\tau\in[0,\tau_{\max}]} \int_0^\tau \hat{C}_1(\tau - s)^{-1/2} \|f(u_1, v_1) - f(u_2, v_2)\|_{\mathcal{L}_2(\Gamma)} \,\mathrm{d}s \\
& + \max_{\tau\in[0,\tau_{\max}]} \int_0^\tau \hat{C}_2(\tau - s)^{-1/2} \|f(u_1, v_1) - f(u_2, v_2)\|_{\mathcal{L}_2(\Gamma)} \,\mathrm{d}s \\
& + \max_{\tau\in[0,\tau_{\max}]} \int_0^\tau \hat{C}_2(\tau - s)^{-1/2} \|q(u_1, v_1) - q(u_2, v_2)\|_{\mathcal{L}_2(\Gamma)} \,\mathrm{d}s \\
& \leq \max_{\tau\in[0,\tau_{\max}]} \int_0^\tau \tilde{C}_{11}(\tau - s)^{-1/2} \left(\|u_1 - u_2\|_{\mathcal{H}^1(\Gamma)} + \|v_1 - v_2\|_{\mathcal{H}^1(\Gamma)}\right) \mathrm{d}s \\
& + \max_{\tau\in[0,\tau_{\max}]} \int_0^\tau \tilde{C}_{21}(\tau - s)^{-1/2} \left(\|u_1 - u_2\|_{\mathcal{H}^1(\Gamma)} + \|v_1 - v_2\|_{\mathcal{H}^1(\Gamma)}\right) \mathrm{d}s \\
& + \max_{\tau\in[0,\tau_{\max}]} \int_0^\tau \tilde{C}_2(\tau - s)^{-1/2} \left(\|u_1 - u_2\|_{\mathcal{H}^1(\Gamma)} + \|v_1 - v_2\|_{\mathcal{H}^1(\Gamma)}\right) \mathrm{d}s \\
& \leq \left((\tilde{C}_{12} + \tilde{C}_{21}) + \tilde{C}_2\right) \tau_{\max}^{1/2} \max_{\tau\in[0,\tau_{\max}]} \left(\|u_1 - u_2\|_{\mathcal{H}^1(\Gamma)} + \|v_1 - v_2\|_{\mathcal{H}^1(\Gamma)}\right) \\
& = \left((\tilde{C}_{12} + \tilde{C}_{21}) + \tilde{C}_2\right) \tau_{\max}^{1/2} \|\mathbf{u}_1 - \mathbf{u}_2\|_X
\end{aligned}$$

and thus choosing $\tau_{\max}$ such that

$$\tau_{\max}^{1/2} < \frac{1}{(\tilde{C}_{12} + \tilde{C}_{21}) + \tilde{C}_2}$$

yields that $\mathbf{T}$ is a contraction. This shows we have a solution in local time, but in fact since the region $\mathcal{A}$ is a trapping region, it means that the solutions $u, v$ are bounded in the $\mathcal{H}^1$-norm for every $\tau$, and from this follows that the existence of solutions is global in time. This is proven by repeating the above argument in a recursive fashion, and each time new initial conditions are chosen by the mapping $(u(\tau_{\max}), v(\tau_{\max})) \mapsto (u_0, v_0)$.

□

## Supplementary Text 1.4 Proof of Theorem 2

The proof is based on the dynamics of the trajectories within the region $\mathcal{A}$ of the $(u, v)$ state space defined as follows:

$$\mathcal{A} := \left\{ \begin{pmatrix} u \\ v \end{pmatrix} \geq \begin{pmatrix} 0 \\ 0 \end{pmatrix} : u + v \leq \min\left(c_{\max}, m\right) \right\}, \quad \text{where } m = \frac{V_0}{a}. \tag{16}$$

The same analysis for another choice of $f$ has been conducted by Röger and Rätz (see Supplementary Reference [39]). To prove this we study the trajectories at the sides of this region. The region $\mathcal{A}$ is a trapping region if the following three conditions are satisfied for positive states $u, v > 0$:

1. $\partial_\tau u = f(u, v) > 0$ at $u = 0$, $0 \leq v \leq \min(c_{\max}, m)$,
2. $\partial_\tau v = -f(u, v) + q(u, v) > 0$ at $v = 0$, $0 \leq u \leq \min(c_{\max}, m)$,
3. $\partial_\tau (u + v) = q(u, v) < 0$ at $u + v = \min\{c_{\max}, m\}$, $0 \leq u, 0 \leq v$.

For the homogeneous system, the functions $f$ and $q$ are defined as follows

$$f(u, v) = c_2 v - u + u^2 v,$$
$$q(u, v) = c_1 a(c_{\max} - (u + v))(m - (u + v)) - c_{-1} v.$$

The first condition yields:

$$\partial_\tau u\big|_{u=0} = f(0, v) = c_2 v > 0 \forall v > 0.$$

The second condition yields:

$$\partial_\tau v\big|_{v=0} = -f(u, 0) + q(u, 0) = u + c_1 a \underbrace{(c_{\max} - u)}_{>0} \underbrace{(m - u)}_{>0} > 0 \forall u \in \mathcal{A}.$$

The third condition yields:

$$\partial_\tau (u + v)\big|_{u+v=\min\{c_{\max}, m\}} = q(u, v)\big|_{u+v=\min\{c_{\max}, m\}} = -c_{-1} v < 0 \forall v > 0.$$

□

### Supplementary Text 1.4.1 Corollary 1 following from Theorem 2

The result follows directly from theorem 3.1 in Supplementary Reference [38]. The trapping region above shows that the states satisfy the condition known as "*positivity*", i.e. $\partial_\tau v\big|_{u=0}, \partial_\tau v\big|_{u=0} > 0$. Also, they need to satisfy what is called "*mass control*" meaning that $\partial_\tau (u + v)$ should be bounded at all times. But this follows from the fact that $q$ is bounded, i.e.

$$\partial_\tau (u + v) = q(u, v) \leq c_1 c_{\max} V_0 < \infty.$$

Also, the states are non-negative $u, v \geq 0$ due to the positivity in combination with the positive initial conditions and we have a uniform upper bound on both states due to mass conservation.

□

## Supplementary Text 1.5 Diffusion driven instability in the limit $D \to \infty$

We reduce the complexity of the original system in (4) in the article by considering the limit $D \to \infty$. This implies that the cytosolic concentration of GDI-bound Cdc42 is approximated as being homogeneous motivated by the fact that the internal diffusion $D$ is much faster compared to the diffusion in the membrane. In this case, the mass conservation property is described by the *non-local functional* $V[u+v]$ below

$$V[u+v] = V_0 - \frac{1}{|\Omega|}\int_{\Gamma}(u+v)\,\mathrm{d}s \tag{17}$$

and the RD-system in (4) in the article gets reduced to the following two-state system:

$$\begin{aligned}\frac{\partial u}{\partial t} &= \Delta u + \gamma f(u,v),\\ \frac{\partial v}{\partial t} &= d\Delta v + \gamma(-f(u,v) + q(u,v,V[u+v])).\end{aligned} \tag{18}$$

This system (Supplementary Equation (17) and Supplementary Equation (18)) was first described in Röger and Rätz (Supplementary Reference [40]) and we will use the same notation as the one introduced in this work here. Now, the stability analysis concerns both the homogeneous system without spatial effects and the inhomogeneous system accounting for spatial effects. In the former case, the non-local functional is transformed to the non-local function $V_1(u+v)$ defined as follows:

$$\begin{aligned} V_1(u+v) &= V_0 - a(u+v),\\ &\text{where}\\ a &= \frac{|\Gamma|}{|\Omega|}.\end{aligned} \tag{19}$$

Note that in our case the domain $\Omega$ is the unit ball after the non-dimensionalisation and $\Gamma$ is the unit sphere. Consequently, the parameter $a$ (Supplementary Equation (19)) has the value

$$a = \frac{|\Gamma|}{|\Omega|} = \frac{4\pi}{4\pi/3} = 3.$$

Now, let $(u^\star, v^\star, V^\star)$ be a steady state of the system in Supplementary Equation (18) and let $f_u$, $f_v$, $q_u$, $q_v$, $q_V$ and $V_1' = -a$ denote the partial derivatives evaluated at this steady state. Then, the stability of the homogeneous system is given by the following conditions:

$$0 > f_u - f_v + q_v + q_V V_1^{'}, \tag{20}$$

$$0 < f_u(q_v + q_V V_1^{'}) - f_v(q_u + q_V V_1^{'}), \tag{21}$$

where the partial derivatives of the function $q$ have negative signs, i.e. $q_u, q_v, (q_V V_1^{'}) < 0$.

As in Proposition 3.1 by Röger and Rätz (Supplementary Reference [40]), it is possible to obtain symmetry breaking in two ways provided that the above conditions (Supplementary Equation (20) and Supplementary Equation (21)) are satisfied. The first way is by the classic diffusion-driven instability proposed by Alan Turing (Supplementary Reference [44]) corresponding to what we will refer to as the classic Turing conditions:

$$f_u q_v - f_v q_u \geq 0, \tag{22}$$
$$d f_u - f_v + q_v > 0, \tag{23}$$
$$Q := (d f_u - f_v + q_v)^2 - 4d(f_u q_v - f_v q_u) \geq 0. \tag{24}$$

Here, the eigenvalues $\lambda_{\pm}$ of the membrane bound Laplace operator $\Delta_\Gamma$ often referred to as the *wave numbers* are given by the following equation:

$$\lambda_{\pm} = \frac{1}{2d}(d f_u - f_v + q_v \pm \sqrt{Q}). \tag{25}$$

In fact, two conclusions can be drawn from the classic conditions (Supplementary Equation (20) and Supplementary Equation (23)). Firstly, the diffusion ratio must satisfy $d > 1$ implying that the state $v$ diffuses faster than $u$. Secondly, the sign of the diagonal elements of the Jacobian matrix $J$ must be opposite and more precisely we must have that

$$J(1,1) > 0 \text{ and } J(2,2) < 0.$$

In linear stability analysis, the Jacobian matrix for a general system with reaction terms determined by $f$ and $g$ consists of the partial derivatives of $f$ and $g$ with respect to the states $u$ and $v$ where these derivatives are evaluated at the steady-state $(u^*, v^*)$ of interest. As a consequence of the latter, it follows that the elements of the Jacobian matrix for our system at hand must have the following signs (Supplementary Reference [35, Fig 2.6 Page 88]):

$$J = \begin{pmatrix} f_u & f_v \\ -f_u + q_u + q_V V_1' & -f_v + q_v + q_V V_1' \end{pmatrix} \text{ with signs } \propto \begin{cases} \begin{pmatrix} + & - \\ + & - \end{pmatrix} \\ \\ \begin{pmatrix} + & + \\ - & - \end{pmatrix} \end{cases}. \tag{26}$$

The other way by which symmetry breaking can be achieved which we will call *non-classic* Turing conditions are formulated as follows:

$$f_u q_v - f_v q_u < 0, \tag{27}$$
$$\lambda_+ > 0. \tag{28}$$

In the article, we state sufficient conditions for characterising a steady state that gives rise to symmetry breaking by means of these two cases of diffusion driven instability. Also, we show that our model will always have either 0, 2, 4 or 6 steady states and in fact there will always be at least one steady state in the trapping region $\mathcal{A}$ in Supplementary Equation (16). These results are summarised in Theorem 3 in the article and numerically we calculate the steady state that can give rise to any of the two cases of Turing instability using our theoretical result in Theorem 3. Given such a candidate steady state, it is the conditions formulated above that are checked numerically by evaluating the partial derivatives of $f$ and $q$ in these conditions at the steady-state of interest. The details of how this is done will be presented in the next part of this document (Supplementary Text 2.1). Before this is done, we prove Theorem 3 in the article stating the existence of steady-states and the characterisation of a steady state at which the system undergoes diffusion driven instability.

## Supplementary Text 1.6 Proof of Theorem 3

We divide the proof of Theorem 3 into two parts. Firstly, we prove the number of steady states of the system. Secondly, we prove the existence of at least one steady state in the region $\mathcal{A}$ and then we derive a sufficient condition allowing for diffusion-driven instability.

### Supplementary Text 1.6.1 Existence of a steady state to the homogeneous system

Recall that a steady state is a solution $(u, v) = (u^*, v^*)$ to the following two equations:

$$\begin{aligned} f(u, v) &= c_2 v - u + u^2 v = 0, \\ q(u, v) &= c_1(V_0 - a(u + v))(c_{\max} - (u + v)) - c_{-1} v = 0. \end{aligned}$$

The first equation is satisfied for all $v$ of the form

$$v = \frac{u}{c_2 + u^2}$$

which can be inserted into the second equation, resulting in

$$c_1 \left( V_0 - a \left( u + \frac{u}{c_2 + u^2} \right) \right) \left( c_{\max} - \left( u + \frac{u}{c_2 + u^2} \right) \right) - c_{-1} \frac{u}{c_2 + u^2} = 0 \tag{29}$$

$$\Longrightarrow c_1 V_0 (c_2 + u^2) - c_1 a \left( u(c_2 + u^2) + u \right) \left( c_{\max} - \left( u + \frac{u}{c_2 + u^2} \right) \right) - c_{-1} u = 0$$

$$\Longleftrightarrow$$

$$\boxed{p(u) = c_1 V_0 (c_2 + u^2)^2 - c_1 a \left( u(c_2 + u^2) + u \right) \left[ c_{\max}(c_2 + u^2) - u(c_2 + u^2) + u \right] - c_{-1} u (c_2 + u^2)} = 0 \tag{30}$$

which is a $6^{\text{th}}$-degree polynomial in $u$ denoted $p(u)$. This polynomial can be simplified and written as follows:

$$\begin{aligned} p(u) &= c_1 V_0 c_2^2 \\ &-u \left( a c_1 c_2 c_{\max}(1 + c_2) + c_2 c_{-1} \right) \\ &+u^2 (a c_1 (1 + c^2)^2 + 2 c_1 c_2 V_0) \\ &-u^3 (a c_1 c_{\max}(1 + 2c_2) + c_{-1}) \\ &+u^4 (2 a c_1 (1 + c_2) + c_1 V_0) \\ &-u^5 (a c_1 c_{\max}) \\ &+u^6 (a c_1). \end{aligned}$$

The solutions to $p(u) = 0$ for the polynomial $p$ in Supplementary Equation (30) subject to one additional condition (Supplementary Equation (16)) which will be introduced in the subsequent section (Supplementary Text 1.6.2) correspond to the steady-states of the original system.

Notice, that since all parameters in the expression above are positive, the coefficients of the polynomial are all positive in the case of even degree terms, and negative in the case of odd degree terms. We can therefore refer to Descartes' rule of signs, to conclude that the polynomial has no negative real roots. We see this by observing that $p(-u)$ has 0 sign changes between consecutive pairs of terms, and therefore there are no negative real roots. Being a $6^{\text{th}}$-degree polynomial, there can therefore be 0, 2, 4 or 6 positive real roots to the polynomial. However, it is worth emphasising that we are only interested in the roots of the polynomial $p$ that lie in

the trapping region $\mathcal{A}$ in (9) in the article. Therefore, we analyse whether the system will have any steady-states in this region or not.

First, observe that $p(0) = c_1 V_0 c_2^2 > 0$ *if we assume that* $c_1 > 0$. Next, we let $u_1$ be defined such that

$$u_1 + \frac{u_1}{c_2 + u_1^2} = \min\left(c_{\max}, \frac{V_0}{a}\right)$$

where $u_1 \geq 0$ which implies that $u_1$ is located on the boundary "$u + v = \min(c_{\max}, V_0/a)$" of the trapping region $\mathcal{A}$ in Supplementary Equation (16). Given this value, we investigate the polynomial $p(u)$ in Supplementary Equation (30) evaluated at $u = u_1$. By re-writing Supplementary Equation (30) according to Supplementary Equation (29) it follows that the value of $p(u_1)$ is proportional to

$$c_1\left(V_0 - a\left(u_1 + \frac{u_1}{c_2 + u_1^2}\right)\right)\left(c_{\max} - \left(u_1 + \frac{u_1}{c_2 + u_1^2}\right)\right) - c_{-1}\frac{u_1}{c_2 + u_1^2}$$

and we see that one of the two factors in the parentheses in the first term will be zero. Thus, the remaining term (stemming from the expression for $p(u_1)$) is "$-((c_{-1}u_1)/(c_2 + u_1^2)) \leq 0$" *if we assume that* $c_{-1} > 0$. Therefore, the sign of the polynomial at $u = u_1$ will also be negative, i.e. $p(u_1) \leq 0$. By the intermediate value theorem, we can now deduce that at least once $p$ is zero on the interval $(0, u_1)$ implying that there is at least one steady-state in the interval $u^* \in (0, \min(c_{\max}, V_0/a))$. Note that this condition is sufficient under the assumption that $c_1, c_{-1} > 0$, and if both or either one of $c_1$ and $c_{-1}$ are negative then it follows that both $p(0)$ and $p(u_1)$ could have the same signs implying that it is not possible to deduce the number of steady states in the interval in this case.

□

### Supplementary Text 1.6.2 Characterisation of the steady-states enabling diffusion-driven instability

We seek a homogeneous steady state $(u^*, v^*) \in \mathcal{A}$ satisfying the following equations:

$$f(u, v) = f(u^*, v^*) = 0, \tag{31}$$
$$q(u, v) = q(u^*, v^*) = 0. \tag{32}$$

The first nullcline in Supplementary Equation (31) implies that the steady state of interest lies on the curve

$$v(u) = \frac{u}{c_2 + u^2} \tag{33}$$

and we are now interested in a $u$-component such that $\Phi(u) := q(u, v(u)) = 0$. We next differentiate $v(u)$ with respect to $u$ and find that

$$v'(u) = \frac{c_2 - u^2}{(c_2 + u^2)^2} = \frac{(\sqrt{c_2} - u)(\sqrt{c_2} + u)}{(c_2 + u^2)^2}$$

which has a positive root[3] at $u_0 = \sqrt{c_2}$, where $v(u_0) = 1/(2\sqrt{c_2})$. We also find that $v'(u) < 0$ for all $u > u_0$ and that $v'(u) > 0$ for all $u < u_0$. It is the switch of signs around the critical point $u_0$ which we will use to characterise the desired steady-states.

Since, $f(u, v) = c_2 v - u + u^2 v$, the corresponding partial derivatives are

[3]The critical point $u_0$ is a local max-point of the function $v(u)$.

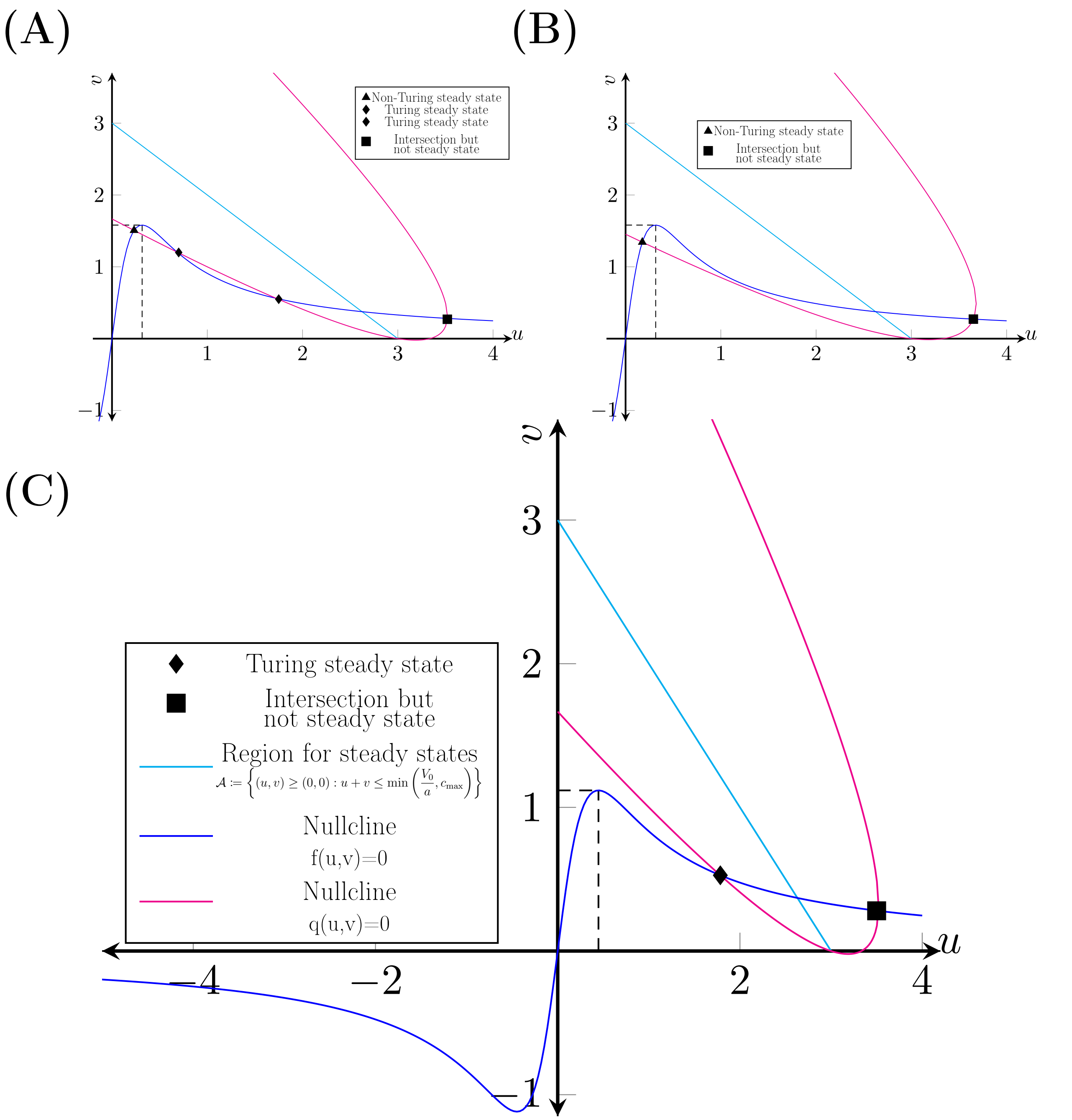


**Supplementary Figure 1: Steady States**. The steady states of the system are located below within the region $\mathcal{A}$ in the first quadrant (the cyan curve). The intersections of two curves define the steady states: the nullcline corresponding to the activation-inactivation reaction $f(u,v) = c_2 v - u + u^2 v$ (the blue curve) and the nullcline corresponding to the transfer of inactive Cdc42 between the cytosol and the membrane $q(u,v) = c_1 \left(V_0 - a(u+v)\right)\left(c_{\max} - (u+v)\right) - c_{-1} v$ (the magenta curve). The number of steady states determined by the intersections of the two nullclines are visualised for three cases. **(A)** *Four steady states* $(c_{-1}, c_2) = (0.30, 0.10)$: one non-Turing steady state ▲, two Turing steady states ♦ and one intersection but not a steady state ■. **(B)** *Two steady states* $(c_{-1}, c_2) = (0.30, 0.20)$: one non-Turing steady state ▲, and one intersection but not a steady state ■. **(C)** *Two steady states* $(c_{-1}, c_2) = (0.20, 0.20)$: one Turing steady state ♦, and one intersection but not a steady state ■. The other parameters are: $V_0 = 10$, $a = 3$, $c_{\max} = 3$ and $c_1 = 0.05$.

$$f_v = c_2 + u^2 > 0$$
$$f_u = 2uv - 1$$

and since these terms are evaluated at the steady state we can substitute $v(u)$ in Supplementary Equation (33) into the latter expression for $f_u$ above to obtain the following.

$$\begin{aligned} f_u &= 2uv - 1 \\ &= 2u\left(\frac{u}{c_2+u^2}\right) - 1 \\ &= \frac{2u^2 - (c_2+u^2)}{c_2+u^2} \\ &= \frac{u^2 - c_2}{c_2+u^2} \\ &= -\left(\underbrace{\frac{c_2-u^2}{(c_2+u^2)^2}}_{=v'(u)}\right)\underbrace{(c_2+u^2)}_{=f_v} \\ &= -v'(u)f_v \end{aligned}$$

Now, the partial derivatives are evaluated at the steady-state $(u^*, v^*)$ which can be characterised based on the sign of the derivative $v_u$ at the steady-state. It is known that a steady-state allowing for diffusion-driven instability has a Jacobian matrix with elements with particular signs (Supplementary Equation (26)). As a consequence of the negativity of the partial derivatives of $q$, the only way to obtain opposite signs of the diagonal elements $J(1,1)$ and $J(2,2)$ is if the following holds:

$$\begin{aligned} f_v &= c_2 + u^2 > 0, \\ f_u &= -v'(u)f_v > 0. \end{aligned} \tag{34}$$

It is clear that $f_v > 0$ however $f_u > 0$ implies that $u^* > u_0$ for the critical point $u_0 = \sqrt{c_2}$. This follows from the fact that we have that $v'(u) < 0 \forall u > u_0$, and $-v'(u) > 0 \forall u > u_0$. Combining this with the fact that the steady-states should be located withing the region $\mathcal{A}$ in Supplementary Equation (16) yields the following characterisation of the desired steady-state

$$u^* \in (u_0, \min(c_{\max}, m)). \tag{35}$$

In fact, this requirement indicate that the signs of the Jacobian must satisfy the lower of the two cases in terms of the signs of the elements in Supplementary Equation (26).

□

# Supplementary Text 2 Numerical implementations

This section consists of three parts: (**1**) A pseudo-code for the visualisation of the parameter space (Fig 2) giving rise to diffusion-driven instability, (**2**) The implementation of the FD-FEM based algorithm for solving the full system in (4) in the article and (**3**) An empirical pole-detection algorithm which terminates the simulation when the pole is formed is presented.

## Supplementary Text 2.1 Numerical mapping of the parameter space

The numerical mapping of the parameter space consists of three steps. For the sake of simplicity, assume that the $(c_{-1}, c_2)$-space[4] is to be calculated. Then, the initial step is to discretise both the $c_{-1}$- and $c_2$-line into a finite number $N \in \mathbb{N}_+$ of nodes. Using this partitioning, allocate memory for the solution matrix $C \in \mathbb{R}_+^{N\times N}$, i.e. $C \leftarrow \mathbf{0}^{N\times N}$. Then loop over the nodes in the partitioning and do the following two steps for all nodes:

1. Calculate the steady states $(u^*, v^*) \in \mathcal{A}$ with the current parameters $(c_{-1}, c_2)$,
2. Check whether any of the calculated steady states $(u^*, v^*) \in \mathcal{A}$ satisfy the desired conditions for diffusion-driven instability:
   (a) **Classic case**: Check the conditions Supplementary Equation (20), Supplementary Equation (21), Supplementary Equation (22), Supplementary Equation (23) and Supplementary Equation (24). If they are satisfied for a certain set of parameters, assign $C(i,j) \leftarrow 1.0$ for the specific indices $i, j \in \{1, \dots, N\}$ of interest,
   (b) **Non-classic case**: Check the conditions in Supplementary Equation (20), Supplementary Equation (21), Supplementary Equation (27) and Supplementary Equation (28). If they are satisfied, assign $C(i,j) \leftarrow 0.5$ for the specific indices $i, j \in \{1, \dots, N\}$ of interest.

Depending on the specific case, a matrix with the value 1.0 for all parameters giving rise to classic diffusion-driven instability is obtained or a corresponding matrix with the value 0.5 in the non-classic case.

To calculate the steady-states, we used a Newton-iteration with a given initial value. To this end, we take the function $v$ in Supplementary Equation (33) corresponding to the nullcline $f(u,v) = 0$. Similarly, we re-write the nullcline $q(u,v) = 0$ as a function of $u$ in order to obtain the following two segments.:

$$v_{q,1}(u) = \frac{1}{2}\left(K + \sqrt{K^2 - 4L}\right), \tag{36}$$

$$v_{q,2}(u) = \frac{1}{2}\left(K - \sqrt{K^2 - 4L}\right), \tag{37}$$

where,

$$A = c_{\max} - u,$$
$$B = V_0 - a\,u,$$
$$K = \frac{1}{a}\left(aA + B + \frac{c_{-1}}{c_1}\right),$$
$$L = \frac{AB}{a}.$$

Now, given a start-guess $u = u_0$, we solve the equations $v(u) - v_{q,1}(u) = 0$ and $v(u) - v_{q,2}(u) = 0$ numerically using the function `fzero` in Matlab (Supplementary Reference [34]). In order to make sure that the solver actually finds the steady-states, we take a large number of initial values (in fact, we have used 50 start guesses) in the interval $[u_0, \min(c_{\max}, m)]$ and we control that each value $u^*$ that the solver converges to satisfies the equation $q\left(u^*, \frac{u^*}{c_2 + (u^*)^2}\right) = 0$. If so, the value $(u^*, v(u^*))$ is a steady state and it is consequently saved.

[4] The $(c_{-1}, c_2)$-space is generated completely analogously.

Lastly, given the steady state we can check the conditions involving the partial derivatives. The partial derivatives for our model are the following:

$$
\begin{aligned}
f_u &= 2uv - 1, \\
f_v &= c_2 + u^2, \\
q_u &= (-c_1 a)(m - (u+v)), \\
q_v &= q_u - c_{-1}, \\
q_V &= c_1(c_{\max} - (u+v)), \\
V' &= -a.
\end{aligned}
$$

## Supplementary Text 2.2 Numerical solutions to the RD-model of cell polarisation

The numerical implementation of the solution to the problem is divided into three parts. Firstly, we set up the problem by formulating the equations of the model and the corresponding domains to the various equations. It is worth emphasising that in the analysis, it is assumed that the cytosolic diffusion goes to infinity, i.e. $D \to \infty$ while this is not the case for the numerical solutions. Moreover, as the variables in the PDE-problem are $\mathbf{x} \in \mathbb{R}^3$ corresponding to the *spatial* dimension and $t \in \mathbb{R}_+$ corresponding to time, two discretisations corresponding to these variables are required. Firstly, a *Finite Element Method* (*FEM*) is implemented corresponding to the spatial discretisation. Secondly, a *Finite Difference* (*FD*)-method is implemented corresponding to the discretisation in time. These two discretisations are presented subsequently after the numerical setting is introduced.

### Supplementary Text 2.2.1 Introduction to the numerical implementation: Setting up the problem

The numerical solution to the original RD problem (Eq (4) in the article) accounts for the domains in the problem. These are the cytosol $\Omega$ corresponding to the interior of the spherical cell and the cell-membrane $\Gamma$ being its surface (Supplementary Figure 2).

The domain in Supplementary Equation (38) and interfaces in Supplementary Equation (39) are important to define as the various equations and boundary conditions are based on the spatial description:

$$\Omega := \left\{\mathbf{x} \in \mathbb{R}^3 : ||\mathbf{x}||_2 < 1\right\}, \tag{38}$$

$$\Gamma := \left\{\mathbf{x} \in \mathbb{R}^3 : ||\mathbf{x}||_2 = 1\right\}. \tag{39}$$

Note that the domains are the unit sphere and its surface which correspond to the geometric description of the dimensionless model.

Now, given the definition of the spatial domain $\Omega$ the formulation of the problem is the following. In the membrane $\Gamma$ the active $u$ and inactive $v$ form undergo activation-inactivation reactions determined by the function $f(u,v) = c_2 v - u + u^2 v$ and diffusion. The interface $\Gamma$ has a flux of the inactive membrane bound component $v$ from the membrane to the cytosol, a flux of the GDI-bound cytosolic component $V$ from the cytosol to the membrane and no flux of the active membrane bound component $u$. The flux over the membrane is determined by the function $q(u,v,V) = c_1 V(c_{\max} - (u+v)) - c_1 v$ and it is translated into a contribution to the reaction term for the inactive membrane-bound species $v$ and a non-homogeneous Neumann

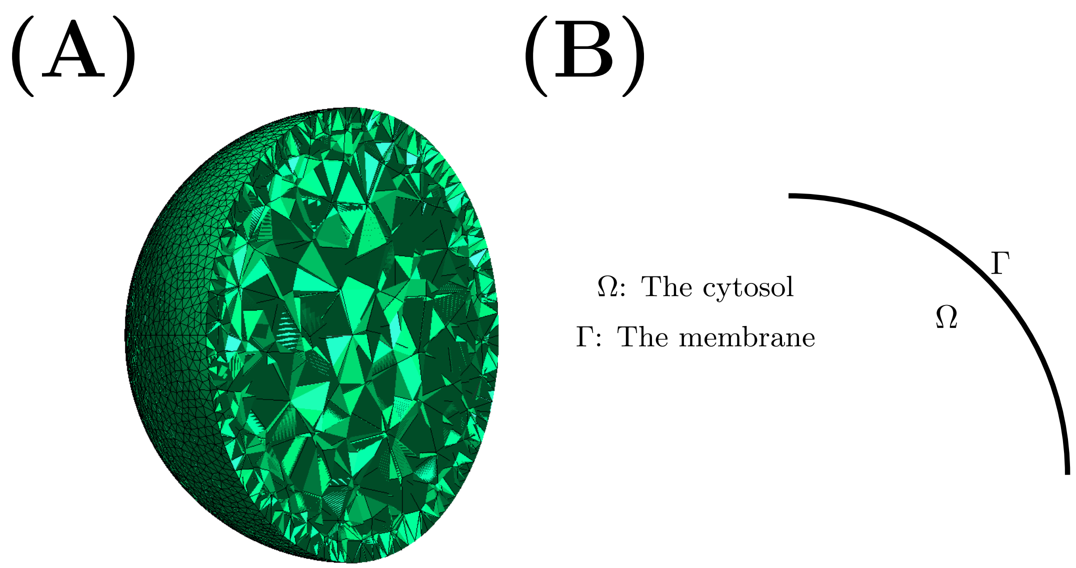


**Supplementary Figure 2: The detailed spatial domain**. (**A**) *Spatial mesh for the numerical solution.* The discretisation of the spatial domain represented as a mesh. The mesh is non-uniformly discretised in the sense that the node-density is higher at the membrane than in the cytosol. (**B**) *Geometric domain.* By letting the membrane thickness shrink to zero, the geometric description is simplified to one domain $\Omega$ corresponding to the cytosol and one boundary $\Gamma$ corresponding to the membrane.

boundary condition for the cytosolic component $V$ at $\Gamma$. Lastly, in the cytosol $\Omega$, the GDI-bound component $V$ undergoes diffusion. Using these equations and boundary conditions it is possible to formulate the detailed model of Cdc42 activation as in Supplementary Equation (40):

$$
\begin{aligned}
&\left.\frac{\partial V}{\partial t} = D\Delta V\right\}\mathbf{x}\in\Omega,\ \tau\in\mathbb{R}_+,\\
&\left.\begin{aligned}-D\left[(\nabla V)^T\cdot\mathbf{n}\right] &= \gamma\left\{c_1 V\left(c_{\max}-(u+v)\right)-c_{-1}v\right\}\\ &= \gamma q(u,v,V)\end{aligned}\right\}\mathbf{x}\in\Gamma,\ \tau\in\mathbb{R}_+,\\
&\left.\begin{aligned}\frac{\partial u}{\partial \tau} &= \gamma\left(c_2 v - u + u^2 v\right)+\Delta u\\ &= \gamma f(u,v)+\Delta u\\ \frac{\partial v}{\partial \tau} &= \gamma\left(-f(u,v)+q(u,v,V)\right)+d\Delta v\end{aligned}\right\}\mathbf{x}\in\Gamma,\ \tau\in\mathbb{R}_+.
\end{aligned}
\tag{40}
$$

The various notations above are the following: $\nabla = \begin{pmatrix}\frac{\partial}{\partial x}\\ \frac{\partial}{\partial y}\\ \frac{\partial}{\partial z}\end{pmatrix}$ is the gradient operator, "$^T$" is the transpose operator, $\mathbf{n}\in\Omega\subset\mathbb{R}^3$ is the *outward normal* at a specific position on a given surface and $\Delta = \frac{\partial^2}{\partial x^2}+\frac{\partial^2}{\partial y^2}+\frac{\partial^2}{\partial z^2}$ is the Laplace operator. In order to solve the problem in Supplementary Equation (40) numerically, the spatial domain must be discretised.

The spatial discretisation of the domain is corresponds to a *mesh* (Supplementary Figure

2A). As the interesting parts of the model regards the reactions and diffusion of the membrane-bound species $u$ and $v$ it is advantageous from a computational view to use a non-uniform mesh. More precisely, we have implemented a mesh with higher node density close to the membrane and a low node density in the interior of the cell. To generate the mesh over the domain, we have used the three dimensional finite element mesh generator Gmsh (Supplementary Reference [12]). Given the generated mesh, it is possible to numerically solve the problem in Supplementary Equation (40) using the finite element method.

For the FD- and FEM-implementations, the computing platform FEniCS (Supplementary References [19, 21, 22, 25, 26, 23, 28, 36, 24, 29, 42, 4, 6, 32, 37, 14, 17, 41, 5, 2, 30, 33, 31, 20, 7, 3, 1, 15, 16]) has been used. In FEniCS, it suffices to provide the so called *variational formulation* of the problem in Supplementary Equation (40) which is a reformulation of the problem. The approximation to this problem is then obtained by projecting the variational formulation onto a space of piece wise continuous functions or in our case a test function space of piece-wise linear functions. In the next chapter, we will merely present the derivation of the variational formulation regarding the spatial discretisation, and after that we will present the finite difference implementation for the temporal discretisation based on this variational formulation.

### Supplementary Text 2.2.2 An implementation of the Finite Element Method in space

Define the following function space for *test functions* $\phi$ on the domain $\Omega$:

$$H^1(\Omega) := \left\{\text{Functions } \phi : \|\phi\|^2_{\mathcal{L}_2(\Omega)} + \|\phi'\|^2_{\mathcal{L}_2(\Omega)} < \infty\right\}. \tag{41}$$

As is standard in the formulation of the variational formulation, we multiply the three PDEs in Supplementary Equation (40) with three test functions $\phi_1, \phi_2, \phi_3 \in H^1(\Omega)$ and integrate over the domain, $\Omega$:

$$\begin{aligned}
\int_\Gamma \frac{\partial u}{\partial \tau}\phi_1(\mathbf{x})\mathrm{d}\mathbf{x} &= \int_\Gamma \Delta u \phi_1(\mathbf{x})\mathrm{d}\mathbf{x} + \gamma \int_\Gamma f(u,v)\phi_1(\mathbf{x})\mathrm{d}\mathbf{x},\\
\int_\Gamma \frac{\partial v}{\partial \tau}\phi_2(\mathbf{x})\mathrm{d}\mathbf{x} &= d\int_\Gamma \Delta v \phi_2(\mathbf{x})\mathrm{d}\mathbf{x} + \gamma \int_\Gamma \left(-f(u,v) + q(u,v,V)\right)\phi_2(\mathbf{x})\mathrm{d}\mathbf{x},\\
\int_\Omega \frac{\partial V}{\partial \tau}\phi_3(\mathbf{x})\mathrm{d}\mathbf{x} &= \int_\Omega D\,\Delta V \phi_3(\mathbf{x})\mathrm{d}\mathbf{x}.
\end{aligned}$$

Now, to rewrite the diffusion terms, we will use *Green's first identity* (Supplementary Reference [27])

$$\int_\Omega v\Delta u \mathrm{d}\mathbf{x} = \int_\Gamma v\,((\nabla u)^T \cdot \mathbf{n})\mathrm{d}s - \int_\Omega (\nabla v)^T \cdot (\nabla u)\mathrm{d}\mathbf{x}$$

where $\nabla = \begin{pmatrix}\frac{\partial}{\partial x}\\ \frac{\partial}{\partial y}\\ \frac{\partial}{\partial z}\end{pmatrix}$ is the gradient, $\mathbf{n} \in \mathbb{R}^3$ is the outward normal and $^T$ is the transpose operator.

Using this identity on the diffusion terms above one obtains

$$\int_\Gamma \frac{\partial u}{\partial \tau}\phi_1(\mathbf{x})\mathrm{d}\mathbf{x} = -\int_\Gamma (\nabla\phi_1(\mathbf{x}))^T\cdot(\nabla u)\mathrm{d}\mathbf{x} + \gamma\int_\Gamma f(u,v)\phi_1(\mathbf{x})\mathrm{d}\mathbf{x},$$
$$\int_\Gamma \frac{\partial v}{\partial \tau}\phi_2(\mathbf{x})\mathrm{d}\mathbf{x} = -d\int_\Gamma (\nabla\phi_2(\mathbf{x}))^T\cdot(\nabla v)\mathrm{d}\mathbf{x} + \gamma\int_\Gamma (-f(u,v)+q(u,v,V))\phi_2(\mathbf{x})\mathrm{d}\mathbf{x},$$
$$\int_\Omega \frac{\partial V}{\partial \tau}\phi_3(\mathbf{x})\mathrm{d}\mathbf{x} = D\int_\Gamma \phi_3(\mathbf{x})\,((\nabla V)^T\cdot\mathbf{n})\mathrm{d}s - \int_\Omega D(\nabla\phi_3(\mathbf{x}))^T\cdot(\nabla V)\mathrm{d}\mathbf{x},$$

and simplifying the above yields the following:

$$\int_\Gamma \frac{\partial u}{\partial \tau}\phi_1(\mathbf{x})\mathrm{d}\mathbf{x} = -\int_\Gamma (\nabla\phi_1(\mathbf{x}))^T\cdot(\nabla u)\mathrm{d}\mathbf{x} + \gamma\int_\Gamma f(u,v)\phi_1(\mathbf{x})\mathrm{d}\mathbf{x},$$
$$\int_\Gamma \frac{\partial v}{\partial \tau}\phi_2(\mathbf{x})\mathrm{d}\mathbf{x} = \gamma\int_\Gamma q(u,v,V)\phi_2(\mathbf{x})\mathrm{d}s - d\int_\Gamma (\nabla\phi_2(\mathbf{x}))^T\cdot(\nabla v)\mathrm{d}\mathbf{x} - \gamma\int_\Gamma f(u,v)\phi_2(\mathbf{x})\mathrm{d}\mathbf{x},$$
$$\int_\Omega \frac{\partial V}{\partial \tau}\phi_3(\mathbf{x})\mathrm{d}\mathbf{x} = -\gamma\int_\Gamma q(u,v,V)\phi_3(\mathbf{x})\mathrm{d}s - \int_\Omega D(\nabla\phi_3(\mathbf{x}))^T\cdot(\nabla V)\mathrm{d}\mathbf{x}.$$

Lastly, moving all terms in the right hand side to the left hand side and adding all three equations yields the *variational formulation* of the detailed problem in Supplementary Equation (40) formulated below:

Find $u(\tau), v(\tau), V(\tau) \in H^1(\Omega)$ for a fixed $\tau \in \mathbb{R}_+$ such that

$$F(u(\tau),v(\tau),V(\tau),\phi_1,\phi_2,\phi_3) = 0 \;\; \forall\phi_1,\phi_2,\phi_3 \in H^1(\Omega) \tag{42}$$

where

$$\begin{aligned}
F(u(\tau),v(\tau),V(\tau),\phi_1,\phi_2,\phi_3) =& \\
&\int_\Gamma \frac{\partial u}{\partial \tau}\phi_1(\mathbf{x})\mathrm{d}\mathbf{x} + \int_\Gamma (\nabla\phi_1(\mathbf{x}))^T\cdot(\nabla u(\tau))\mathrm{d}\mathbf{x}\\
&+\int_\Gamma \frac{\partial v}{\partial \tau}\phi_2(\mathbf{x})\mathrm{d}\mathbf{x} + d\int_\Gamma (\nabla\phi_2(\mathbf{x}))^T\cdot(\nabla v(\tau))\mathrm{d}\mathbf{x}\\
&+\gamma\int_\Gamma f(u(\tau),v(\tau))(\phi_2(\mathbf{x})-\phi_1(\mathbf{x}))\mathrm{d}\mathbf{x}\\
&+\int_\Omega \frac{\partial V}{\partial \tau}\phi_3(\mathbf{x})\mathrm{d}\mathbf{x} + D\int_\Omega (\nabla\phi_3(\mathbf{x}))^T\cdot(\nabla V(\tau))\mathrm{d}\mathbf{x}\\
&+\gamma\int_\Gamma q(u(\tau),v(\tau),V(\tau))(\phi_3(\mathbf{x})-\phi_2(\mathbf{x}))\mathrm{d}s.
\end{aligned}$$

The numerical solution of the problem in Supplementary Equation (42) is written as linear combinations of piece-wise continuous basis functions of order 1, i.e. linear functions. Assume that a spatial discretisation (Supplementary Figure 2A) is given and denote this by $\tau_h$. To describe the finite element method, denote the space of piece-wise linear functions on $\tau_h$ by $\mathfrak{H}(\Omega)$. These tetrahedron-like functions take the value one on each node in the grid (Supplementary Figure 2A) and they take the value zero on the neighbouring nodes. Then, the finite-element solution $\mathfrak{u}(\tau), \mathfrak{v}(\tau), \mathfrak{V}(\tau) \in \mathfrak{H}(\Omega)$ for a fixed $\tau \in \mathbb{R}_+$ to the variational formulation in Supplementary Equation (42) consists of the orthogonal projections of $u(\tau), v(\tau), V(\tau) \in H^1(\Omega)$ onto $\mathfrak{H}(\Omega)$ for a fixed $\tau \in \mathbb{R}_+$. Here, we mean projection in the sense of the classic $\mathcal{L}_2$-*inner product*:

$$\langle f, g\rangle = \int_\Omega f(\mathbf{x})g(\mathbf{x})\,\mathrm{d}\mathbf{x}.$$

It is this variational formulation in Supplementary Equation (42) that is solved in FEniCS approximately using the finite element method just described. Subsequently, the time derivatives will be approximated using a finite difference scheme.

### Supplementary Text 2.2.3 An implementation of the Finite Difference Method in time

For the implementation of the finite difference scheme in order to solve the problem in Supplementary Equation (42) numerically, a *mixed Implicit-Explicit scheme* was implemented. The solution of the ODE problem

$$\frac{\mathrm{d}y}{\mathrm{d}t} = f(y(t))$$

with $f \in \mathcal{C}(\mathbb{R})$ is the function $y \in \mathcal{C}^1(\mathbb{R}_+)$ with the time $t \in \mathbb{R}_+$ as variable. Initially, the general methodology for finite differences involves *discretising* the time-line into various nodes, where the partitioning of the time-line is denoted as follows $\mathcal{T}([0, t_{\max}]) = \{t_0, t_1, t_2, \ldots, t_n, \ldots, t_{\max}\}$. Moreover, denote each of these nodes by $t_n \in \mathbb{R}_+$ for some index $n \in \mathbb{Z}_+$ where the previous node on the discretised time line is denoted $t_{n-1}$. Then, the backward-Euler algorithm for the above problem entails solving solve the following equation iteratively

$$\frac{y(t_n) - y(t_{n-1})}{k_n} = f(y(t_n))$$

where $k_n = t_n - t_{n-1} \in \mathbb{R}_+$ is the so called *step size.* The solution to the above equation corresponds to the solution $y(t_n)$ in the current node given the solution in the previous node $y(t_{n-1})$. Note that both the left and the right hand side in the above equation depend on the solution $y(t_n)$ in the current node which implies that this algorithm is *implicit.* The forward version of the Euler algorithm consists of solving

$$\frac{y(t_n) - y(t_{n-1})}{k_n} = f(y(t_{n-1}))$$

which yields the iterative scheme $y(t_n) = y(t_{n-1}) + k_n f(y(t_{n-1}))$ which is *explicit* and can be solved directly. However, the implicit version is more computationally expensive and has to be solved by using for example Newton's method (Supplementary References [8, 9]). Despite the implicit algorithm being more computationally expensive compared to the forward or explicit version of the Euler scheme for ODEs, the advantage of it is that it is *unconditionally stable* as oppose the the explicit algorithm which is merely *conditionally stable.* The numerical solution of the detailed problem in Supplementary Equation (40) is given by combining the finite element method based on the previous variational formulation in Supplementary Equation (42) with a mixed explicit-implicit Euler finite difference scheme described above, which results in the variational formulation presented in Supplementary Equation (43) below:

$$
\begin{aligned}
&\text{Find } \; u(\tau_n), v(\tau_n), V(\tau_n) \in H^1(\Omega)\\
&\qquad F(u(\tau_n), v(\tau_n), V(\tau_n), u(\tau_{n-1}), v(\tau_{n-1}), V(\tau_{n-1}), \phi_1, \phi_2, \phi_3) = 0 \;\; \forall \phi_1, \phi_2, \phi_3 \in H^1(\Omega)\\
&\qquad\qquad \text{where}\\
&\qquad F(u(\tau_n), v(\tau_n), V(\tau_n), u(\tau_{n-1}), v(\tau_{n-1}), V(\tau_{n-1}), \phi_1, \phi_2, \phi_3) =\\
&\qquad\qquad \int_\Gamma \left(\frac{u(\tau_n) - u(\tau_{n-1})}{\tau_n - \tau_{n-1}}\right)\phi_1(\mathbf{x})\mathrm{d}\mathbf{x} + \int_\Gamma (\nabla\phi_1(\mathbf{x}))^T \cdot (\nabla u(\tau_n))\mathrm{d}\mathbf{x}\\
&\qquad\qquad + \int_\Gamma \left(\frac{v(\tau_n) - v(\tau_{n-1})}{\tau_n - \tau_{n-1}}\right)\phi_2(\mathbf{x})\mathrm{d}\mathbf{x} + d\int_\Gamma (\nabla\phi_2(\mathbf{x}))^T \cdot (\nabla v(\tau_n))\mathrm{d}\mathbf{x}\\
&\qquad\qquad + \gamma\int_\Gamma f(u(\tau_n), v(\tau_n), u(\tau_{n-1}), v(\tau_{n-1}))(\phi_2(\mathbf{x}) - \phi_1(\mathbf{x}))\mathrm{d}\mathbf{x}\\
&\qquad\qquad + \int_\Omega \left(\frac{V(\tau_n) - V(\tau_{n-1})}{\tau_n - \tau_{n-1}}\right)\phi_3(\mathbf{x})\mathrm{d}\mathbf{x} + D\int_\Omega (\nabla\phi_3(\mathbf{x}))^T \cdot (\nabla V(\tau_n))\mathrm{d}\mathbf{x}\\
&\qquad\qquad + \gamma\int_\Gamma q(u(\tau_n), v(\tau_n), u(\tau_{n-1}), v(\tau_{n-1}), V(\tau_{n-1}))(\phi_3(\mathbf{x}) - \phi_2(\mathbf{x}))\mathrm{d}s.
\end{aligned} \tag{43}
$$

The above approach is referred to as an *mixed Implicit-Explicit* approach. This approach is implicit in the sense that the linear terms, i.e. the time derivatives approximated by finite differences and the diffusive terms containing the gradients, are implicit. These will result in the classic mass- and stiffness-matrices for the time derivatives and diffusive terms respectively when the finite element method is implemented. By "trial and error" we found that a *mixed implicit-explicit* methodology for approximating the non-linear reaction terms $f$ and $q$ worked in order to get accurate simulations:

$$
\begin{aligned}
f(u(\tau_n), v(\tau_n), u(\tau_{n-1}), v(\tau_{n-1})) &= c_2 v(\tau_n) - u(\tau_n) + u(\tau_{n-1})^2 v(\tau_{n-1}),\\
q(u(\tau_n), v(\tau_n), u(\tau_{n-1}), v(\tau_{n-1}), V(\tau_{n-1})) &=\\
&c_1 V(\tau_{n-1})\left(c_{\max} - (u(\tau_{n-1}) + v(\tau_{n-1}))\right) - c_{-1} v(\tau_n).
\end{aligned} \tag{44}
$$

As can be seen, all purely linear terms are treated implicitly while the purely non-linear terms are treated explicitly. Furthermore, we implemented an adaptive explicit time-stepping procedure to approximate the time derivatives. The time-stepping procedure is based on calculating the residual "Res $= |F - 0| = |F|$" in Supplementary Equation (43). In each time-step, we calculate a possible maximum time step $\mathrm{d}\tau$ by setting it inversely proportional to Res, i.e.:

$$\delta\tau \leftarrow \frac{\mathrm{TOL}}{\mathrm{Res}}$$

where the tolerance TOL is defined beforehand. We also save the previous time step $\Delta\tau$

$$\Delta\tau \leftarrow (\tau_n - \tau_{n-1})$$

and then the new time step is calculated by using the arithmetic mean according to the following rule:

$$k \leftarrow \min\left(\frac{2 \cdot \Delta\tau \cdot \delta\tau}{\Delta\tau + \delta\tau}, k_{\max}\right).$$

In similarity with the tolerance TOL, another numerical parameter corresponding to the maximum step length $k_{\max}$ is defined beforehand. The above rule for choosing the step length $k$

in time renders the implemented algortihm *adaptive* in time. This algorithm takes long time steps when we have a small error, i.e. a small residual, when Suplementary Equation (43) is solved and it takes short time steps when a large error is obtained in the calculations.

To test the validity of our implementation, a test-problem was constructed. To this end, we solved the homogeneous ODE-problem

$$\begin{aligned}\frac{\partial u}{\partial \tau} &= \gamma\left(c_2 v - u + u^2 v\right)\\ &= \gamma f(u,v)\\ \frac{\partial v}{\partial \tau} &= \gamma\left(f(u,v) + [c_1(V_0 - a(u+v))(c_{\max} - (u+v)) - c_{-1}v]\right)\\ &= \gamma(-f(u,v) + q(u,v))\end{aligned}$$

with trivial initial conditions

$$u_0 = v_0 = 0.$$

Then, the spatial averages of the PDE-solutions of Supplementary Equation 43 given by

$$\overline{u}(\tau) = \frac{1}{|\Gamma|}\int_\Gamma u(\mathbf{x},\tau)\ \mathrm{d}s \ \ \& \ \ \overline{v}(\tau) = \frac{1}{|\Gamma|}\int_\Gamma v(\mathbf{x},\tau)\ \mathrm{d}s$$

were compared with the ODE-solutions of the homogeneous problem. Again, the initial conditions of $u$ and $v$ in the PDE-setting were set to 0 everywhere on the membrane $\Gamma$ while the initial condition of $V$ was set to $V(\mathbf{x},0) = V_0\ \forall \mathbf{x} \in \Omega$. The spatial averages $(\overline{u}(\tau), \overline{v}(\tau))$ were virtually equal to to the ODE-solutions $(u(\tau), v(\tau))$ when the mixed implementation in Supplementary Equation (44) of the reaction terms was used (Supplementary Figure 3) which validates our implementation.

It is interesting to note that the non-linearities of $f$ and $q$ are evaluated at the previous time node and thereby the algorithm is explicit with respect to the “complicated terms”. A consequence of this is that a large value of the relative diffusion $d$ will render the solution algorithm relatively more implicit (and thus more stable) while a large value of the reaction strength $\gamma$ will render the algorithm relatively more explicit (and thereby less stable). An advantage of the above implementation is that is faster to solve compared to a purely implicit algorithm, however a small time-step $k_n = \tau_n - \tau_{n-1}$ is required to ensure stability. Moreover, the initial conditions for the above time stepping procedure were set to

$$u(x_i,\tau_0) = u(x_i,0) = u^\star + \epsilon_{1,i},\ \ v(x_i,\tau_0) = v(x_i,0) = v^\star + \epsilon_{2,i}\ \text{ and }\ V(x_i,\tau_0) = V(x_i,0) = V^\star + \epsilon_{3,i}$$

for all spatial nodes $x_i, i = 1,\ldots,N$ where $N \in \mathbb{N}_+$ is the total number of nodes. Above, we set the initial conditions for all spatial nodes to the steady-state value $(u^*, v^*, V^*)$ at which diffusion-driven instability occurs plus a noise term $\epsilon_{k,i} \in \mathcal{N}(0,\sigma)$, $k \in \{1,2,3\}$ which is normally distributed with zero mean and standard deviation $\sigma$. For the implementation, we have implemented a noise term of $\sigma = 0.1$.

The presented simulations correspond to the numerical solutions of the problem in Supplementary Equation (40) given by this combined approach. In summary, our methodology entails applying the FEM on the variational formulation in Supplementary Equation (43) repeatedly while advancing in time using FDs corresponding to a mixed implicit-explicit Euler scheme with adaptive time-stepping. Thus, given a spatial disretisation in terms of a mesh (Supplementary Figure 2B), a partitioning of the time-line $\mathcal{T}([0,\tau_{\max}]) = \{\tau_0, \tau_1, \tau_2, \ldots, \tau_n, \ldots, \tau_{\max}\}$ and initial

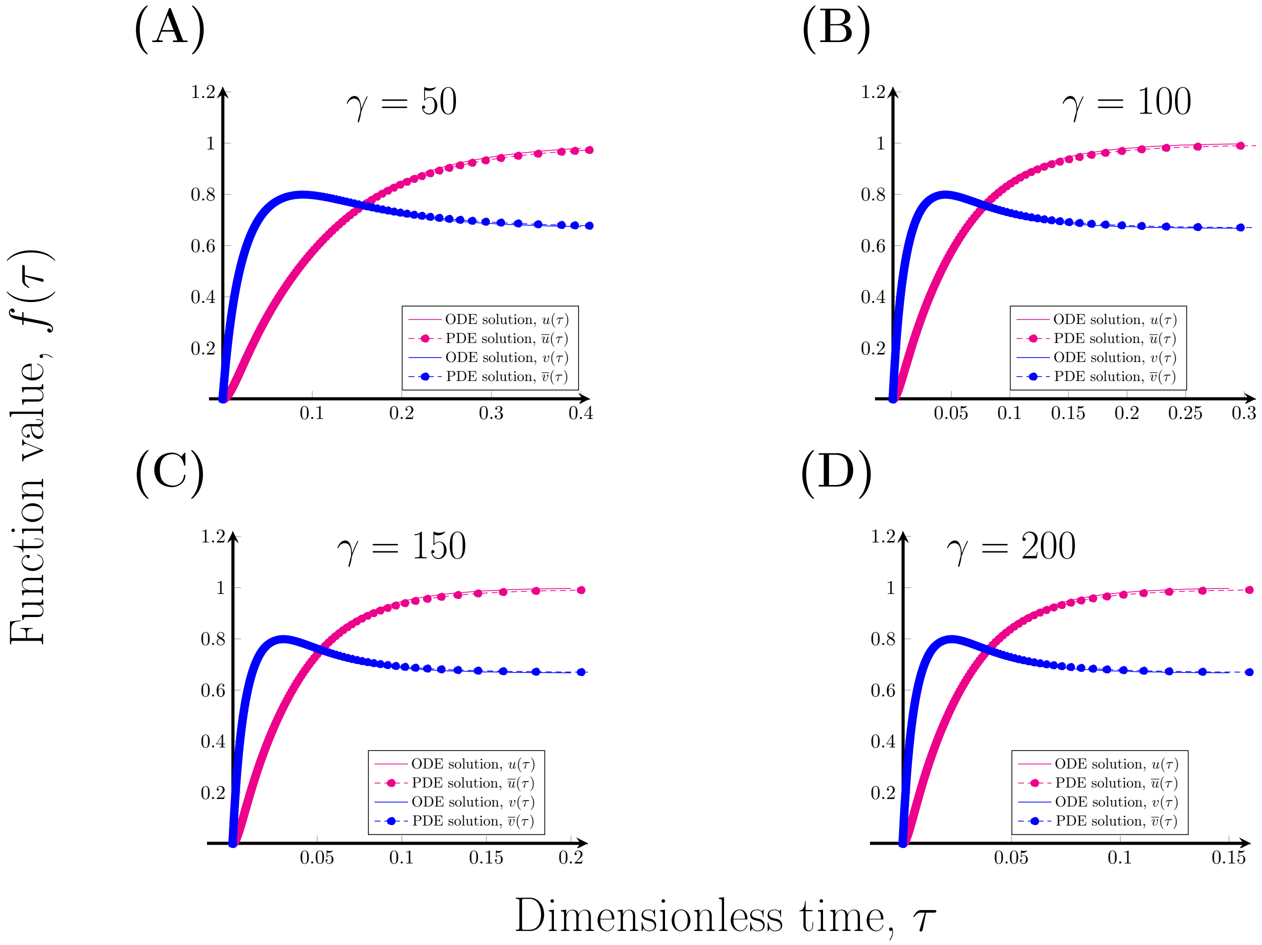


**Supplementary Figure 3: Validation of the FEM-FD implementation**. The states are plotted over time where the active state corresponds to the blue graph and the magenta graph to the inactive state. The ODE solutions $u, v$ are represented withe whole lines while the spatial averages of the PDE solutions $\overline{u}, \overline{v}$ are represented by the dashed lines. The comparison is made in four cases corresponding to **(A)** $\gamma = 50$, **(B)** $\gamma = 100$, **(C)** $\gamma = 150$, and **(D)** $\gamma = 200$. The other parameters used in the simulations are: $c_1 = 0.05$, $c_{-1} = 0.10$, $c_1 = 0.50$, $D = 1000$, $V_0 = 6.0$ and $c_{\max} = 3.0$.

conditions $\{\mathfrak{u}_0, \mathfrak{v}_0, \mathfrak{V}_0\} = \{\mathfrak{u}(\tau_0), \mathfrak{v}(\tau_0), \mathfrak{V}(\tau_0)\} \in \mathfrak{H}$ corresponding to the initial concentration profiles of the three states it is possible to solve the problem numerically using the finite element method based on Supplementary Equation (43) in order to obtain the finite element solutions in each time node in $\mathcal{T}([0, \tau_{\max}])$. Regarding the initial conditions $\{\mathfrak{u}_0, \mathfrak{v}_0, \mathfrak{V}_0\} \in \mathfrak{H}$, we have initiated the three states inhomogeneously, as described above, in their respective domains (i.e. $\Omega$ for $\mathfrak{V}$ and $\Gamma$ for $\mathfrak{u}$ and $\mathfrak{v}$) with a small perturbation around each steady state value.

## Supplementary Text 2.3 Empirical pole-recognition algorithm

Two pole properties that we are especially interested in are pole size and time to polarisation. These properties are obtained by studying the concentration profile of active Cdc42. It is often quite easy to visually verify that a concentration profile is polarised, but to quantitatively determine when a pole arises and its extent is not trivial. We have defined mathematical condition for pole, such that a pole is reached when the concentration profile of the active Cdc42 is approximately constant, i.e. $\partial u/\partial t \approx 0$, and that the maximum concentration differs significantly from the minimum. Numerically, this entails approximating the time derivative with a finite difference and then set two numerical tolerances for when these conditions are deemed to be satisfied. In an attempt to make pole-recognition more consistent, we have developed and implemented a pole-recognition algorithm. The algorithm uses tolerance parameters that have been empirically calibrated in order to reach some agreement between visual and numerical polarisation. The consistency in pole-recognition thus heavily relies on using fixed tolerance parameter values after an initial calibration phase. The algorithm checks the new discrete finite element concentration of active Cdc42, $\mathfrak{u}(\tau_n)$ for every discrete time $\tau_n$ in the time-stepping. The main steps of the algorithm are outlined in pseudo-code below where we use $U^n$ to denote the array of all *surface* nodal values of $\mathfrak{u}(\tau_n)$. We let $U_i^n$ denote an arbitrary element of $U^n$. The empirical tolerance parameters are all denoted TOL with some index.

**Algorithm 1** Empirical pole-recognition (performed at discrete time $\tau_n$)

*TEST 1: Check if concentration profile has not changed much*
**if** $|(U_i^n - U_i^{n-1})/(\tau_n - \tau_{n-1})| < \mathrm{TOL}_{zero}$ for all $i$ **then**
  *TEST 2: Check if concentration profile has binary character*
  Compute $U_{max} = \max_i(U_i^n)$ and $U_{min} = \min_i(U_i^n)$
  Set $N_{max} = 0$ and $N_{min} = 0$
  **for all** $U_i^n \in U^n$ **do**
    **if** $|U_i^n - U_{max}| < \mathrm{TOL}_{max}$ **then**
      $N_{max} = N_{max} + 1$
    **else if** $|U_i^n - U_{min}| < \mathrm{TOL}_{min}$ **then**
      $N_{min} = N_{min} + 1$
    **end if**
  **end for**
  **if** $N_{max} + N_{min} > \mathrm{TOL}_{percent} * \mathrm{length}(U^n)$ **then**
    *POLE DETECTED: Compute and return pole properties*
    Pole size is computed as the pole surface ratio $psr = N_{max}/\mathrm{length}(U^n)$
    Time to polarisation is simply $\tau_n$
    **return** $psr$ and $\tau_n$
  **end if**
**end if**

# Supplementary Text 3 Additional results

In this section, we present additional results from the simulations of cell polarisation. Specifically, the subsequent plots focus on the effect of changing the kinetic rate parameters on the polarisation process. Also, we display the results of increasing the relative diffusion with a relative scale, that is where $u_{\max}$ varies for the different values of $d$.

## Supplementary Text 3.1 Varying the kinetic parameters

Similarly to the effect of changing the relative diffusion $d$ (Fig 4 in the article) and the relative reaction strength $\gamma$ (Fig 6 in the article), the effect of changing the kinetic parameters on the cell polarisation process can be investigated. Therefore, the results of the simulations for different kinetic parameters in the $(c_{-1}, c_2)$-plane (Supplementary Figure 4) and the $(c_1, c_2)$-plane (Supplementary Figure 5) have been visualised.

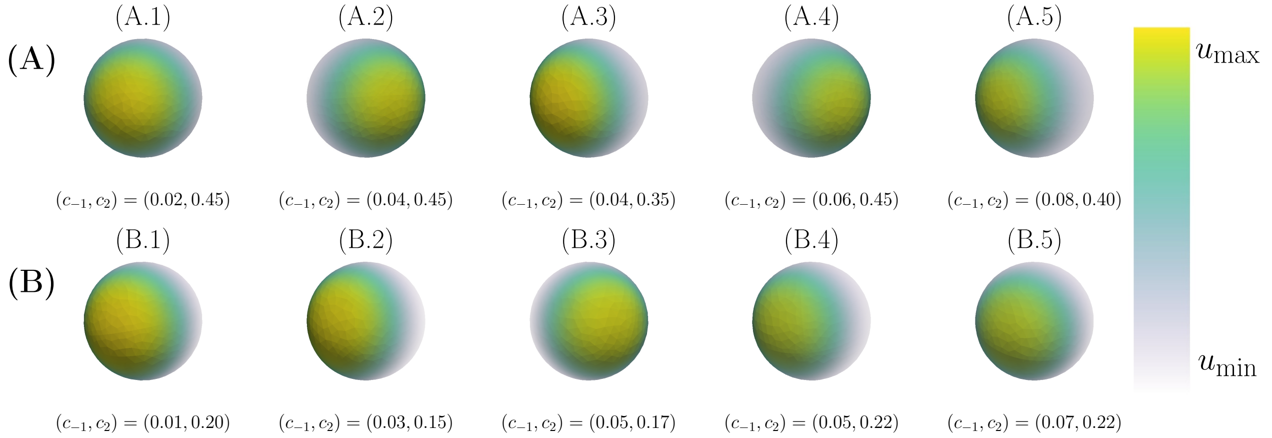


**Supplementary Figure 4: Final patterns for different kinetic parameters in the $(\mathbf{c_{-1}}, \mathbf{c_2})$-plane.** The final patterns for various parameters in the $(c_{-1}, c_2)$-plane are displayed in two cases, namely classic and non-classic diffusion driven instability. In both cases, the final time when the pattern is formed $\tau_{\text{final}}$ and the maximum and minimum concentration of active Cdc42 $u_{\max}$ and $u_{\min}$ are calculated as functions of the kinetic rate parameters. **(A)** *Classic*: (A.1): $(c_{-1}, c_2, \tau_{\text{final}}, u_{\max}, u_{\min}) = (0.02, 0.45, 4.40, 3.77, 0.38)$, (A.2): $(c_{-1}, c_2, \tau_{\text{final}}, u_{\max}, u_{\min}) = (0.04, 0.45, 4.75, 3.64, 0.40)$, (A.3): $(c_{-1}, c_2, \tau_{\text{final}}, u_{\max}, u_{\min}) = (0.04, 0.35, 3.61, 3.83, 0.29)$, (A.4): $(c_{-1}, c_2, \tau_{\text{final}}, u_{\max}, u_{\min}) = (0.06, 0.45, 5.42, 3.55, 0.41)$ and (A.5) $(c_{-1}, c_2, \tau_{\text{final}}, u_{\max}, u_{\min}) = (0.08, 0.40, 4.95, 3.57, 0.37)$. **(B)** *Non-classic*: (B.1): $(c_{-1}, c_2, \tau_{\text{final}}, u_{\max}, u_{\min}) = (0.01, 0.20, 5.50, 4.26, 0.15)$, (B.2): $(c_{-1}, c_2, \tau_{\text{final}}, u_{\max}, u_{\min}) = (0.03, 0.15, 3.38, 4.18, 0.11)$, (B.3): $(c_{-1}, c_2, \tau_{\text{final}}, u_{\max}, u_{\min}) = (0.05, 0.17, 6.61, 4.06, 0.13)$, (B.4): $(c_{-1}, c_2, \tau_{\text{final}}, u_{\max}, u_{\min}) = (0.05, 0.22, 4.70, 3.92, 0.18)$ and (B.5) $(c_{-1}, c_2, \tau_{\text{final}}, u_{\max}, u_{\min}) = (0.07, 0.22, 4.05, 3.91, 0.18)$ . In both cases, the overall parameters are: $c_1 = 0.05$, $V_0 = 6.0$, $c_{\max} = 3.0$, $a = 3$, $d = 10$ and $\gamma = 25$.

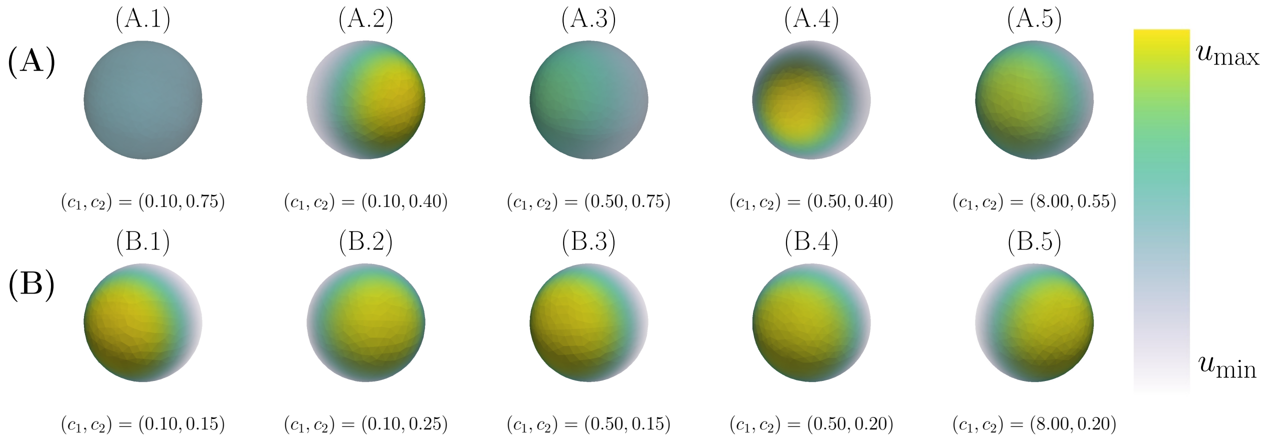


**Supplementary Figure 5: Final patterns for different kinetic parameters in the $(\mathbf{c_1}, \mathbf{c_2})$-plane**. The final patterns for various parameters in the $(c_1, c_2)$-plane are displayed in two cases, namely classic and non-classic diffusion driven instability. In both cases, the final time when the pattern is formed $\tau_{\text{final}}$ and the maximum and minimum concentration of active Cdc42 $u_{\max}$ and $u_{\min}$ are calculated as functions of the kinetic rate parameters. **(A)** *Classic*: (A.1): $(c_1, c_2, \tau_{\text{final}}, u_{\max}, u_{\min}) = (0.10, 0.75, 10.59, 1.54, 1.28)$, (A.2): $(c_1, c_2, \tau_{\text{final}}, u_{\max}, u_{\min}) = (0.10, 0.40, 6.69, 3.65, 0.35)$, (A.3): $(c_1, c_2, \tau_{\text{final}}, u_{\max}, u_{\min}) = (0.50, 0.75, 19.0, 2.65, 0.89)$, (A.4): $(c_1, c_2, \tau_{\text{final}}, u_{\max}, u_{\min}) = (0.50, 0.40, 6.48, 3.59, 0.36)$ and (A.5) $(c_1, c_2, \tau_{\text{final}}, u_{\max}, u_{\min}) = (8, 0.55, 6.51, 3.27, 0.54)$. **(B)** *Non-classic*: (B.1): $(c_1, c_2, \tau_{\text{final}}, u_{\max}, u_{\min}) = (0.10, 0.15, 5.08, 4.02, 0.12)$, (B.2): $(c_1, c_2, \tau_{\text{final}}, u_{\max}, u_{\min}) = (0.10, 0.25, 2.88, 3.88, 0.20)$, (B.3): $(c_1, c_2, \tau_{\text{final}}, u_{\max}, u_{\min}) = (0.50, 0.15, 8.38, 3.93, 0.12)$, (B.4): $(c_1, c_2, \tau_{\text{final}}, u_{\max}, u_{\min}) = (0.50, 0.20, 7.97, 3.87, 0.16)$ and (B.5) $(c_1, c_2, \tau_{\text{final}}, u_{\max}, u_{\min}) = (8, 0.20, 9.04, 3.86, 0.16)$ . In both cases, the overall parameters are: $c_{-1} = 0.05$, $V_0 = 6.0$, $c_{\max} = 3.0$, $a = 3$, $d = 10$ and $\gamma = 25$.

## Supplementary Text 3.2 Effect of increasing diffusion with an absolute scale

Visually, it is not clear that the maximum concentration of active Cdc42 $u_{\max}$ increases with an increasing relative diffusion $d$ in Fig 4 in the article. In this figure, the concentration scale, i.e. the colourbar, is *relative* implying that the value of $u_{\max}$ varies between the different values of the relative diffusion $d$. The advantage with this representation is that it is visually clear that the relative size of the pole decreases with an increasing relative diffusion while the disadvantage is that it is not possible to see the increase in the maximum concentration of active Cdc42. Although, the increase in $u_{\max}$ is presented in the figure caption it is also possible to visually present this effect. To this end, the same figure has been reproduced with an absolute scale on the colour bar (Supplementary Figure 6).

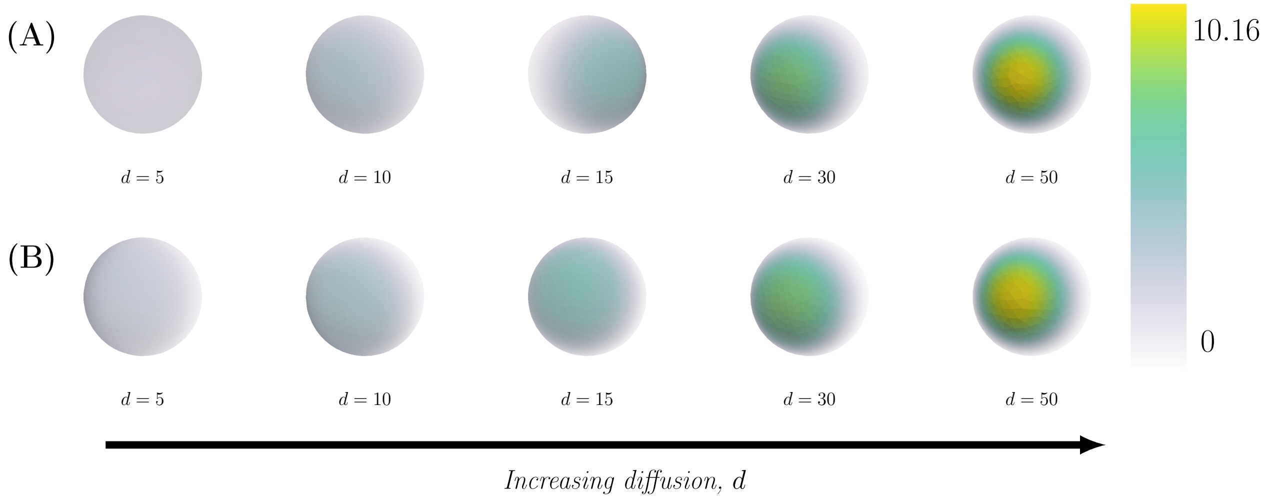


**Supplementary Figure 6: Final patterns for increasing relative diffusion with absolute scale**. The final patterns for increasing relative diffusion $d$ are displayed in two cases, namely classic and non-classic diffusion driven instability. In both cases, the final time when the pattern is formed $\tau_{\text{final}}$ and the maximum and minimum concentrations of active Cdc42 $u_{\max}$ and $u_{\min}$ are calculated as functions of the kinetic rate parameters. **(A)** *Classic*: The overall parameters are $(c_1, c_{-1}, c_2) = (0.05, 0.04, 0.45)$ with specific parameters (from left to right): no pattern is formed for $(d, \tau_{\text{final}}, u_{\max}, u_{\min}) = (5, 15, 20.89, 1.34, 1.11)$, $(d, \tau_{\text{final}}, u_{\max}, u_{\min}) = (10, 4.44, 3.65, 0.40)$, $(d, \tau_{\text{final}}, u_{\max}, u_{\min}) = (15, 3.65, 4.85, 0.30)$, $(d, \tau_{\text{final}}, u_{\max}, u_{\min}) = (30, 2.65, 7.42, 0.20)$ and $(d, \tau_{\text{final}}, u_{\max}, u_{\min}) = (50, 2.17, 9.83, 0.15)$. **(B)** *Non-classic*: The overall parameters are $(c_1, c_{-1}, c_2) = (0.05, 0.03, 0.15)$ with specific parameters (from left to right): $(d, \tau_{\text{final}}, u_{\max}, u_{\min}) = (5, 4.0, 2.77, 0.17)$, $(d, \tau_{\text{final}}, u_{\max}, u_{\min}) = (10, 4.38, 4.18, 0.11)$, $(d, \tau_{\text{final}}, u_{\max}, u_{\min}) = (15, 2.87, 5.29, 0.09)$, $(d, \tau_{\text{final}}, u_{\max}, u_{\min}) = (30, 1.95, 7.77, 0.06)$ and $(d, \tau_{\text{final}}, u_{\max}, u_{\min}) = (50, 1.96, 10.166, 0.04)$. In both cases, the overall parameters are: $V_0 = 6.0$, $c_{\max} = 3.0$, $a = 3$ and $\gamma = 25$.

# Supplementary References

[1] Martin S. Alnæs. *UFL: a Finite Element Form Language*, chapter 17. Springer, 2012.

[2] Martin S. Alnæs, Jan Blechta, Johan Hake, August Johansson, Benjamin Kehlet, Anders Logg, Chris Richardson, Johannes Ring, Marie E. Rognes, and Garth N. Wells. The fenics project version 1.5. *Archive of Numerical Software*, 3(100), 2015.

[3] Martin S. Alnæs, Anders Logg, and Kent-Andre Mardal. *UFC: a Finite Element Code Generation Interface*, chapter 16. Springer, 2012.

[4] Martin S. Alnæs, Anders Logg, Kent-Andre Mardal, Ola Skavhaug, and Hans Petter Langtangen. Unified framework for finite element assembly. *International Journal of Computational Science and Engineering*, 4(4):231–244, 2009.

[5] Martin S. Alnæs, Anders Logg, Kristian B. Ølgaard, Marie E. Rognes, and Garth N. Wells. Unified form language: A domain-specific language for weak formulations of partial differential equations. *ACM Transactions on Mathematical Software*, 40(2), 2014.

[6] Martin S. Alnæs and Kent-Andre Mardal. On the efficiency of symbolic computations combined with code generation for finite element methods. *ACM Transactions on Mathematical Software*, 37(1), 2010.

[7] Martin S. Alnæs and Kent-Andre Mardal. *SyFi and SFC: Symbolic Finite Elements and Form Compilation*, chapter 15. Springer, 2012.

[8] Niclas Andréasson, Anton Evgrafov, Michael Patriksson, Emil Gustavsson, and Magnus Önnheim. *An introduction to continuous optimization: foundations and fundamental algorithms*, volume 28. Studentlitteratur Lund, 2005.

[9] Ward Cheney and David Kincaid. Numerical mathematics and computation, 2004.

[10] Lokenath Debnath, Piotr Mikusinski, et al. *Introduction to Hilbert spaces with applications*. Academic press, 2005.

[11] Gerald B Folland. *Fourier analysis and its applications*, volume 4. American Mathematical Soc., 2009.

[12] Christophe Geuzaine and Jean-François Remacle. Gmsh: A 3-d finite element mesh generator with built-in pre-and post-processing facilities. *International journal for numerical methods in engineering*, 79(11):1309–1331, 2009.

[13] Andrew B Goryachev and Marcin Leda. Many roads to symmetry breaking: molecular mechanisms and theoretical models of yeast cell polarity. *Molecular biology of the cell*, 28(3):370–380, 2017.

[14] Johan Hoffman, Johan Jansson, Rodrigo V. de Abreu, Cem Degirmenci, Niclas Jansson, Kaspar Müller, Murtazo Nazarov, and Jeanette H. Spühler. Unicorn: Parallel adaptive finite element simulation of turbulent flow and fluid-structure interaction for deforming domains and complex geometry. *Computer and Fluids*, in press, 2012.

[15] Johan Hoffman, Johan Jansson, Cem Degirmenci, Niclas Jansson, and Murtazo Nazarov. *Unicorn: a Unified Continuum Mechanics Solver*, chapter 18. Springer, 2012.

[16] Johan Hoffman, Johan Jansson, Niclas Jansson, C. Johnson, and Rodrigo V. de Abreu. *Turbulent Flow and Fluid–structure Interaction*, chapter 28. Springer, 2012.

[17] Niclas Jansson, Johan Jansson, and Johan Hoffman. Framework for massively parallel adaptive finite element computational fluid dynamics on tetrahedral meshes. *SIAM Journal on Scientific Computing*, 34(1):C24–C41, 2012.

[18] Yoshitsugu Kabeya, Tatsuki Kawakami, Atsushi Kosaka, and Hirokazu Ninomiya. Eigenvalues of the laplace-beltrami operator on a large spherical cap under the robin problem. *Kodai Mathematical Journal*, 37(3):620–645, 2014.

[19] Robert C. Kirby. Algorithm 839: Fiat, a new paradigm for computing finite element basis functions. *ACM Transactions on Mathematical Software*, 30(4):502–516, 2004.

[20] Robert C. Kirby. *FIAT: Numerical Construction of Finite Element Basis Functions,*, chapter 13. Springer, 2012.

[21] Robert C. Kirby, Matthew G. Knepley, Anders Logg, and L. Ridgway Scott. Optimizing the evaluation of finite element matrices. *SIAM Journal on Scientific Computing*, 27(3):741–758, 2005.

[22] Robert C. Kirby and Anders Logg. A compiler for variational forms. *ACM Transactions on Mathematical Software*, 32(3), 2006.

[23] Robert C. Kirby and Anders Logg. Efficient compilation of a class of variational forms. *ACM Transactions on Mathematical Software*, 33(3), 2007.

[24] Robert C. Kirby and Anders Logg. Benchmarking domain-specific compiler optimizations for variational forms. *ACM Transactions on Mathematical Software*, 35(2):1–18, 2008.

[25] Robert C. Kirby, Anders Logg, L. Ridgway Scott, and Andy R. Terrel. Topological optimization of the evaluation of finite element matrices. *SIAM Journal on Scientific Computing*, 28(1):224–240, 2006.

[26] Robert C. Kirby and L. Ridgway Scott. Geometric optimization of the evaluation of finite element matrices. *SIAM Journal on Scientific Computing*, 29(2):827–841, 2007.

[27] Stig Larsson and Vidar Thomée. *Partial differential equations with numerical methods*, volume 45. Springer Science & Business Media, 2008.

[28] Anders Logg. Automating the finite element method. *Archives of Computational Methods in Engineering*, 14(2):93–138, 2007.

[29] Anders Logg. Efficient representation of computational meshes. *International Journal of Computational Science and Engineering*, 4(4):283–295, 2009.

[30] Anders Logg, Kent-Andre Mardal, Garth N. Wells, et al. *Automated Solution of Differential Equations by the Finite Element Method.* Springer, 2012.

[31] Anders Logg, Kristian B. Ølgaard, Marie E. Rognes, and Garth N. Wells. *FFC: the FEniCS Form Compiler*, chapter 11. Springer, 2012.

[32] Anders Logg and Garth N. Wells. Dolfin: Automated finite element computing. *ACM Transactions on Mathematical Software*, 37(2), 2010.

[33] Anders Logg, Garth N. Wells, and Johan Hake. *DOLFIN: a C++/Python Finite Element Library*, chapter 10. Springer, 2012.

[34] MATLAB. *version 9.4.0.813654 (R2018a).* The MathWorks Inc., Natick, Massachusetts, 2018.

[35] J. D. Murray. *Mathematical Biology II: Spatial Models and Biomedical Applications*, volume 18 of *Interdisciplinary Applied Mathematics.* Springer New York, 2003.

[36] Kristian B. Ølgaard, Anders Logg, and Garth N. Wells. Automated code generation for discontinuous galerkin methods. *SIAM Journal on Scientific Computing*, 31(2):849–864, 2008.

[37] Kristian B. Ølgaard and Garth N. Wells. Optimisations for quadrature representations of finite element tensors through automated code generation. *ACM Transactions on Mathematical Software*, 37, 2010.

[38] Michel Pierre. Global existence in reaction-diffusion systems with control of mass: a survey. *Milan Journal of Mathematics*, 78(2):417–455, 2010.

[39] Andreas Rätz and Matthias Röger. Turing instabilities in a mathematical model for signaling networks. *Journal of mathematical biology*, 65(6-7):1215–1244, 2012.

[40] Andreas Rätz and Matthias Röger. Symmetry breaking in a bulk–surface reaction–diffusion model for signalling networks. *Nonlinearity*, 27(8):1805, 2014.

[41] Marie E. Rognes, David A. Ham, Colin J. Cotter, and Andrew T. T. McRae. Automating the solution of pdes on the sphere and other manifolds in fenics 1.2. *Geoscientific Model Development*, 6:2099–2119, 2013.

[42] Marie E. Rognes, Robert C. Kirby, and Anders Logg. Efficient assembly of h(div) and h(curl) conforming finite elements. *SIAM Journal on Scientific Computing*, 31(6):4130–4151, 2009.

[43] Vladimir Tulovsky and Lech Papiez. Formula for the fundamental solution of the heat equation on the sphere. *Applied mathematics letters*, 14(7):881–884, 2001.

[44] A. M. Turing. The chemical basis of morphogenesis. *Bulletin of Mathematical Biology*, 52(1):153–197, Jan 1952.
